# Discriminating single-bacterial shape using low-aspect-ratio pores

**DOI:** 10.1038/s41598-017-17443-6

**Published:** 2017-12-12

**Authors:** Makusu Tsutsui, Takeshi Yoshida, Kazumichi Yokota, Hirotoshi Yasaki, Takao Yasui, Akihide Arima, Wataru Tonomura, Kazuki Nagashima, Takeshi Yanagida, Noritada Kaji, Masateru Taniguchi, Takashi Washio, Yoshinobu Baba, Tomoji Kawai

**Affiliations:** 1 The Institute of Scientific and Industrial Research, Osaka University, Ibaraki, Osaka 567-0047 Japan; 2 Department of Applied Chemistry, Graduate School of Engineering and ImPACT Research Center for Advanced Nanobiodevices, Nagoya University, Furo-cho, Chikusa-ku, Nagoya 464-8603 Japan; 3 Japan Science and Technology Agency (JST), PRESTO, 4-1-8 Honcho, Kawaguchi, Saitama 332-0012 Japan; 4 Institute for Materials Chemistry and Engineering, Kyushu University, 6-1 Kasuga-Koen, Kasuga, Fukuoka 816-8580 Japan; 5 Health Research Institute, National Institute of Advanced Industrial Science and Technology (AIST), Takamatsu, 761-0395 Japan

## Abstract

Conventional concepts of resistive pulse analysis is to discriminate particles in liquid by the difference in their size through comparing the amount of ionic current blockage. In sharp contrast, we herein report a proof-of-concept demonstration of the shape sensing capability of solid-state pore sensors by leveraging the synergy between nanopore technology and machine learning. We found ionic current spikes of similar patterns for two bacteria reflecting the closely resembled morphology and size in an ultra-low thickness-to-diameter aspect-ratio pore. We examined the feasibility of a machine learning strategy to pattern-analyse the sub-nanoampere corrugations in each ionic current waveform and identify characteristic electrical signatures signifying nanoscopic differences in the microbial shape, thereby demonstrating discrimination of single-bacterial cells with accuracy up to 90%. This data-analytics-driven microporescopy capability opens new applications of resistive pulse analyses for screening viruses and bacteria by their unique morphologies at a single-particle level.

## Introduction

A resistive pulse method is a versatile technology widely utilized for discriminations of single-bioparticles of variable sizes from blood cells to polynucleotides^1–4^. It probes transient ionic current blockade associated with fast translocation of an individual particle passing through a fluid flow path at which objects of higher volume exclude more ions to render larger pulses. The last decade has witnessed resurgence of this electrical method led by advanced nanotechnology to increase the spatial resolution of the particle analyser by employing ultra-thin membrane materials down to single-atom level. Eventually, it led to the thickness-to-diameter aspect ratio of the conduit to be less than unity^5–12^. In such short channels, the amount of ionic current blockade is anticipated to be determined largely by the local volume of particles occupying the conduit, the characteristic of which would enable rapid 2D-scanning of their morphologies at nanoscale. Meanwhile, although employing thinner membrane would provide higher spatial resolution to the sensor, it also poses additional challenges in interpreting the resistive pulse patterns. This is due to the concomitant increase in the relative significance of the ionic resistance outside the channel (access resistance) that makes the cross-membrane ionic current sensitive to physical features of particles including their fine shapes and the dynamical translocation motions^13^. Accordingly, despite the recent efforts^14,15^, the potential of the envisaged sensing ability remains to be demonstrated.

**Figure 1:**
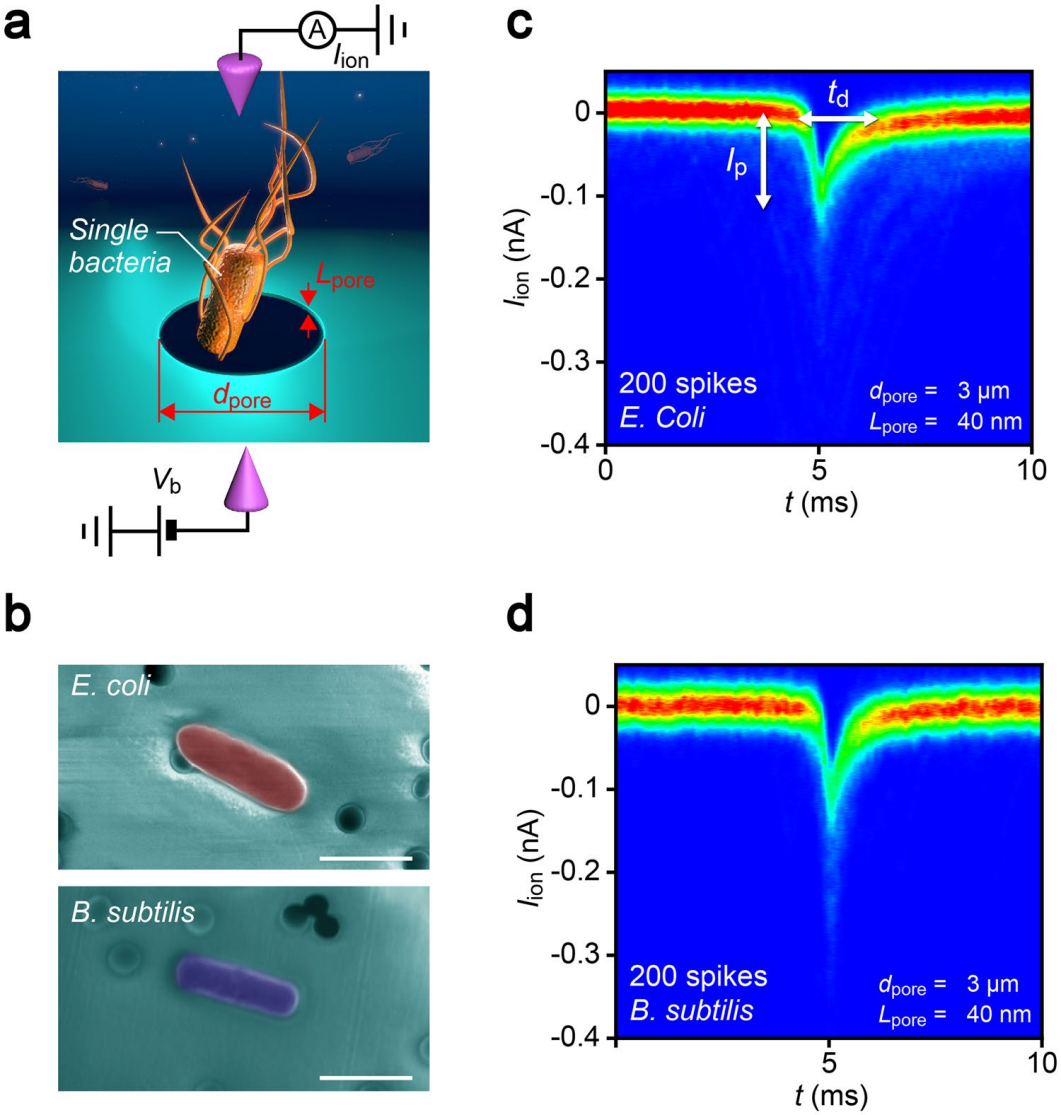
Single-bacteria sensing using low thickness-to-diameter aspect-ratio pore channels. (**a**) Schematic illustration depicting resistive pulse measurements of *Escherichia coli* and *Bacillus subtilis* using a SiN micropore of diameter *d*_pore_ and length *L*_pore_. (**b**) False-colored scanning electron micrographs of *E*. *coli* (top) and *B*. *subtilis* (bottom). Scale bars denote 2 μm. Dark small circles are holes to filter ionic liquid and fix the bacteria on the substrate. (**c**,**d**) The cross-pore ionic current *I*_ion_ versus time *t* two dimensional histograms constructed with 200 ionic current spike signals obtained for (**c**) *E*. *coli* and (**d**) *B*. *subtilis* using a SiN pore with *d*_pore_ = 3.0 μm and *L*_pore_ = 40 nm. *I*_p_ and *t*_d_ denote the pulse height and width, respectively.

We herein report a novel concept of nanopore analysis that leverages the enhanced spatial resolution of ultralow-aspect-ratio pore sensors and machine learning algorithm to elucidate the physics underlies individual resistive pulse wave patterns and discriminate single-particles by its shape instead of the whole volume. The solid-state device consists of a micropore of diameter *d*_pore_ = 3 μm sculpted in a SiN membrane of thickness *L*_pore_ = 40 nm on a Si wafer having aspect ratio *AR* ~ 0.01 (Fig. 1a). We used the pore channel for detections of *Escherichia coli*, potentially pathogenic bacteria ubiquitous in environment^16^, and *Bacillus subtilis* having rod-like shape and micrometer size similar to those of *E*. *coli* (Fig. 1b), by measuring the cross-membrane ionic current *I*_ion_ in PBS buffer (10 times diluted with Milli-Q) under the applied dc voltage *V*_b_ of 0.05 V (Figs 1a and S1). The previous work exploited intermolecular-interaction-derived cell capture dynamics for discriminations of *E*. *coli* from other bacteria using a bionanopore system^17^. In contrast to the immunosensing approach, our aim here is to distinguish the difference in the morphologies of the two bacteria. Ionic current spikes with height *I*_p_ and width *t*_d_ were observed, the parameters of which generally denote the amount of ion blockage and time-of-flight of the negatively-charged single-bacteria electrophoretically passing through the channel (Figs. 1c,d).

## Results and Discussions

A standard approach for single-particle discriminations involve statistical comparisons of *I*_p_ and *t*_d_ variations^18–20^ (Fig. S2). The distributions, however, overlap largely (Fig. S3) whereby failing to discern the two bacteria at discriminability no better than 70% (Fig. S4) due to the biologically different yet physically resembled characteristics including the volume and the surface charge status (zeta potential: −37 mV (*E*. *coli*) and −38 mV (*B*. *subtilis*)). Here, the negative surface charges of the gram negative and positive bacteria come largely from the phospholipids and lipopolysaccharides covering the cell and the phosphates in teichoic acids on peptidoglycan, respectively. On the other hand, while the plots present little difference in the average properties of the bioparticles, we at the same time found a subtle difference in the *I*_ion_ spike line-shapes, the variance of which anticipates not only the size and shape distributions of the two bacteria but also the stochastic nature of the capture dynamics because of the significant contributions of the ion transport in the external regions of the pore^13,21,22^, on the ionic blockage upon particle translocation.

**Figure 2:**
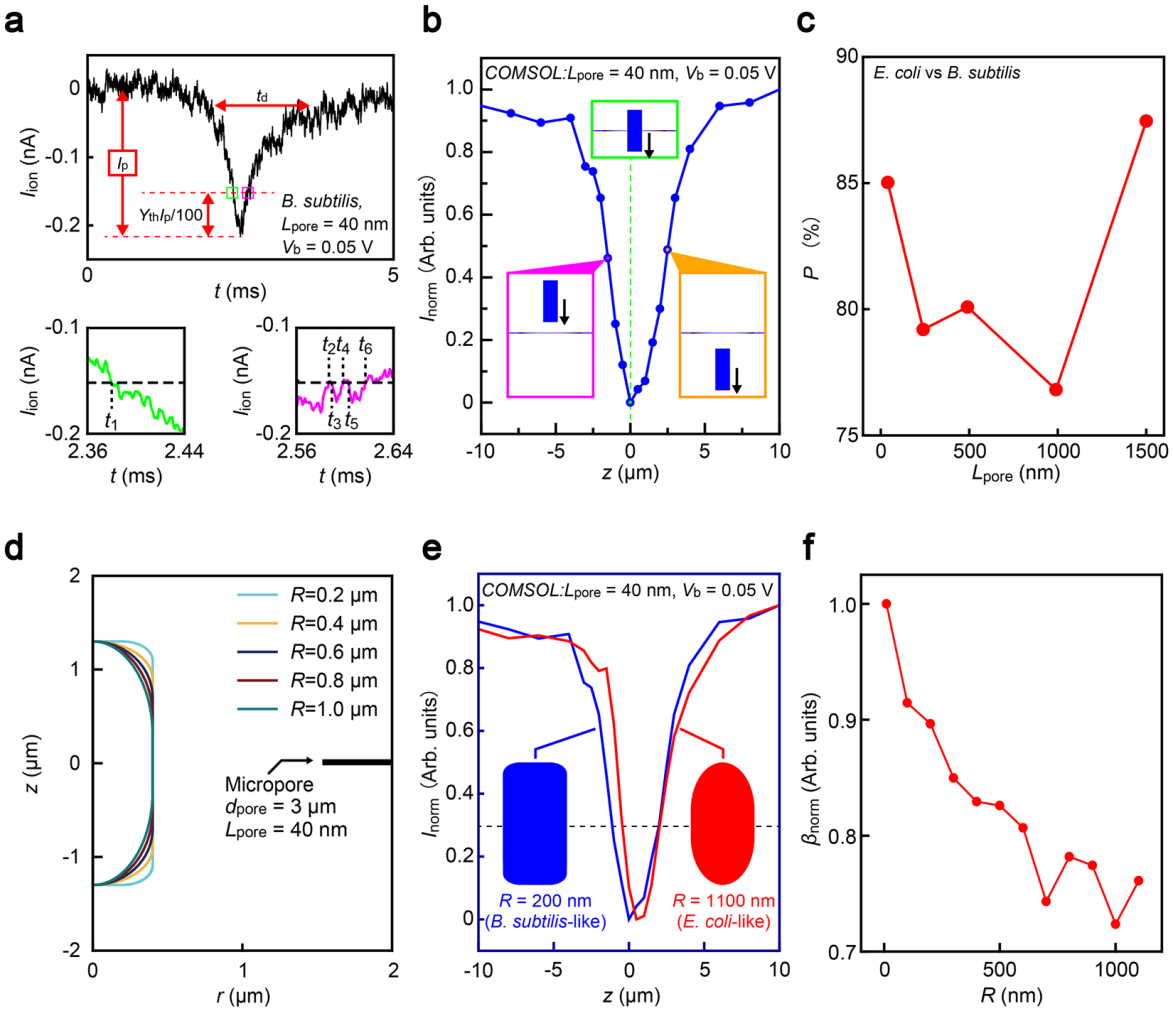
Statistical discriminations of single-bacteria by resistive pulse line shapes. (**a**) Definition of the pulse bluntness *β*_apex_. The time *t*_i_ at which *I*_ion_ crosses the current level *Y*_th_ % above the pulse top is collected. *β*_apex_ is defined as the deviations of *t*_i_. (**b**) Finite element analysis of ionic current blockage in a low-aspect-ratio pore by single-bacteria modelled as a microscale cylinder. (**c**) The accuracy *P*_pre_ for discriminating *E*. *coli* and *B*. *subtilis* through comparing statistical distributions of *β*_apex_ and *I*_p_ via 5-fold cross validation plotted against *L*_pore_. (**d**) A model used for finite element analysis of ionic current blockade by single-microbe. A bacteria-shaped cylinder of diameter 800 nm and length 2.6 μm with different curvature *R* was moved along *z* axis wherein axis-translocation was assumed. (**e**) Normalized resistive pulses deduced from the finite element analysis for two micro-rods with *R* = 200 nm (blue) and 1100 nm (red) that mimic the sharp-edged and rounded shapes of *E*. *coli* and *B*. *subtilis*, respectively. Dotted line is at *I*_norm_ = 0.3 where *β*_apex_ is extracted. The pulse bluntness is lower for cylinders with larger *R*. (**f**) Normalized resistive pulse bluntness *β*_norm_ plotted as a function of the roundness *R* of the cylindrical model of bacteria. *β*_norm_ is deduced from *β*_apex_ of the theoretical *I*_ion_ spikes normalized by that at *R* = 10 nm.

The above results suggest a necessity of a rational approach to first find features relevant to a bacterial morphology in the ionic current signatures to accomplish single-particle shape analysis using a low-aspect-ratio pore sensor. Pattern recognition is an intrinsic ability of animals that spontaneously acts to match perceived information with the memory stored in a brain. Analogously, here we used an artificial intelligence to inspect bacterial traits in the virtually featureless ionic current signatures obtained with a low-aspect-ratio micropore for statistically estimating the respective numbers of *E*. *coli* and *B*. *subtilis*. Specifically, we examined a resistive pulse line-shape analysis in a framework of a machine learning algorithm^23,24^ estimating non-parametric probability density on a feature space consisting of the height *I*_p_ and bluntness *β*_apex_ of the ionic spikes (Figs 2a and S5; see also Supplementary Information 2.1). Noticeably, we found the precision *P*_pre_ exceeding 90% at *Y*_th_ = 30% for the ultra-low aspect-ratio micropore of *AR* ~ 0.01, where *Y*_th_ denotes the current level from the pulse maxima (Fig. S5). Meanwhile, ionic transport calculations based on a Poisson-Nernst-Planck-Navier Stokes model^13^ predict that the bioparticles are not yet getting inside a pore but still moving at the orifice until *I*_ion_ reaches *Y*_th_ = 25% from *I*_p_ (Fig. 2b)^13,21^. This indicates electrophoretic motions of the two bacteria at the pore exterior render no distinctive feature in the *I*_ion_ traces useful for distinguishing the microbes. Conversely, the ionic current profiles below the level, i.e. *β*_apex_ for instance, constitutes electrical fingerprints of single-bacteria wherein local shape of the microorganisms play a dominant role on the ion exclusion inside the pore channel, the finding of which exemplifies the potential of the data-driven analysis to discover physically valid *I*_ion_ characteristics distinct to the analytes.

In order to verify the tomographic capability, we systematically studied the roles of pore geometry on the sensing performance by carrying out the bacterial detections with 3 μm-sized micropores having variable depth from 40 nm to 1500 nm (Fig. S6). The pattern recognition of *I*_ion_ spikes revealed intriguing change in *P* that deteriorated 5-fold upon increasing *L*_pore_ to 990 nm whereas further thickening resulted in recovery of the sensor performance (Fig. 2c). The non-trivial dependence can be interpreted as denoting mutual and competitive contributions of the access resistance and the resistance inside the pore. As the *L*_pore_ is less than the length of the bacteria, longer channels give larger change in the inner-pore resistance during single-bacterial translocation due to the larger amount of ions excluded in the pore thereby offering better sensitivity to the bioparticle volume as denoted by higher *P*_pre_ at *L*_pore_ ≥ 990 nm. Conversely, shorter micropores are expected to show degraded volume sensitivity. Nevertheless, they also possess higher spatial resolution to enable discriminating bacteria by difference in their shape. This is highlighted qualitatively in the feature parameter histograms (Figs S3 and S7) where we found less overlap in the distributions of *β*_apex_ (*I*_p_) with decreasing (increasing) *L*_pore_ whereby suggesting better statistical discriminability by the microbial shape (volume) in thinner (thicker) pore channels. In other words, whereas the ultra-low aspect-ratio pores and thick channels can distinguish the two bacteria with high *P*_pre_ through sensitive detections of the bioparticle shape and volume, respectively, the intermediate range of *AR* leads to deteriorated sensor performance as both the sensing capabilities become poor.

It is of interest to consider more precisely on what affects the apex line-shapes of resistive pulses. We herein explored influence of the bacterial morphologies by running the *I*_ion_ simulations for translocation of bacteria-mimicking rod-shaped objects with various roundness *R* (Fig. 2d; see also S22). The estimations revealed increasing pulse bluntness with *R* (Figs 2e,f and S23), which is in qualitative accordance with the sharper *I*_ion_ spikes obtained for *E*. *coli* having a rounder motif compared to that of *B*. *subtilis* (Fig. 1b). This in turn proves the analytical power of the machine learning approach to computationally discern the bioparticles by the submicrometer-scale body form difference through the *I*_ion_ traces.

Besides the morphologies, the bacterial motility of the flagellated microbes may have caused distinct difference in the capture-to-translocation dynamics whereby facilitated to discern *E*. *coli* and *B*. *subtilis* by the resistive pulse line-shapes. Considering the remote sensing capability of low-aspect-ratio pores to sense the analyte motions nearby the channel entrance, it is indeed worth investigating the possible influence of the swimming ability unique to the bacteria on the ionic signatures^25,26^. We therefore examined resistive pulse measurements for flagellated (wild-type) and non-flagellated (gene fliC inactivated mutant) *E*. *coli* using a 3 μm-sized SiN micropore of *AR* ~ 0.01 (Fig. S8). Contrary to the expectation, however, the results showed so little difference in the ionic spike forms that the machine learning based pattern analysis can discriminate the two by no better than 62%, suggesting no conspicuous roles of flagella on the translocation speed. It can be understood by considering predominant roles of the electrophoretic forces that overwhelms the flagellar motor power to affect the translocation motions.

**Figure 3:**
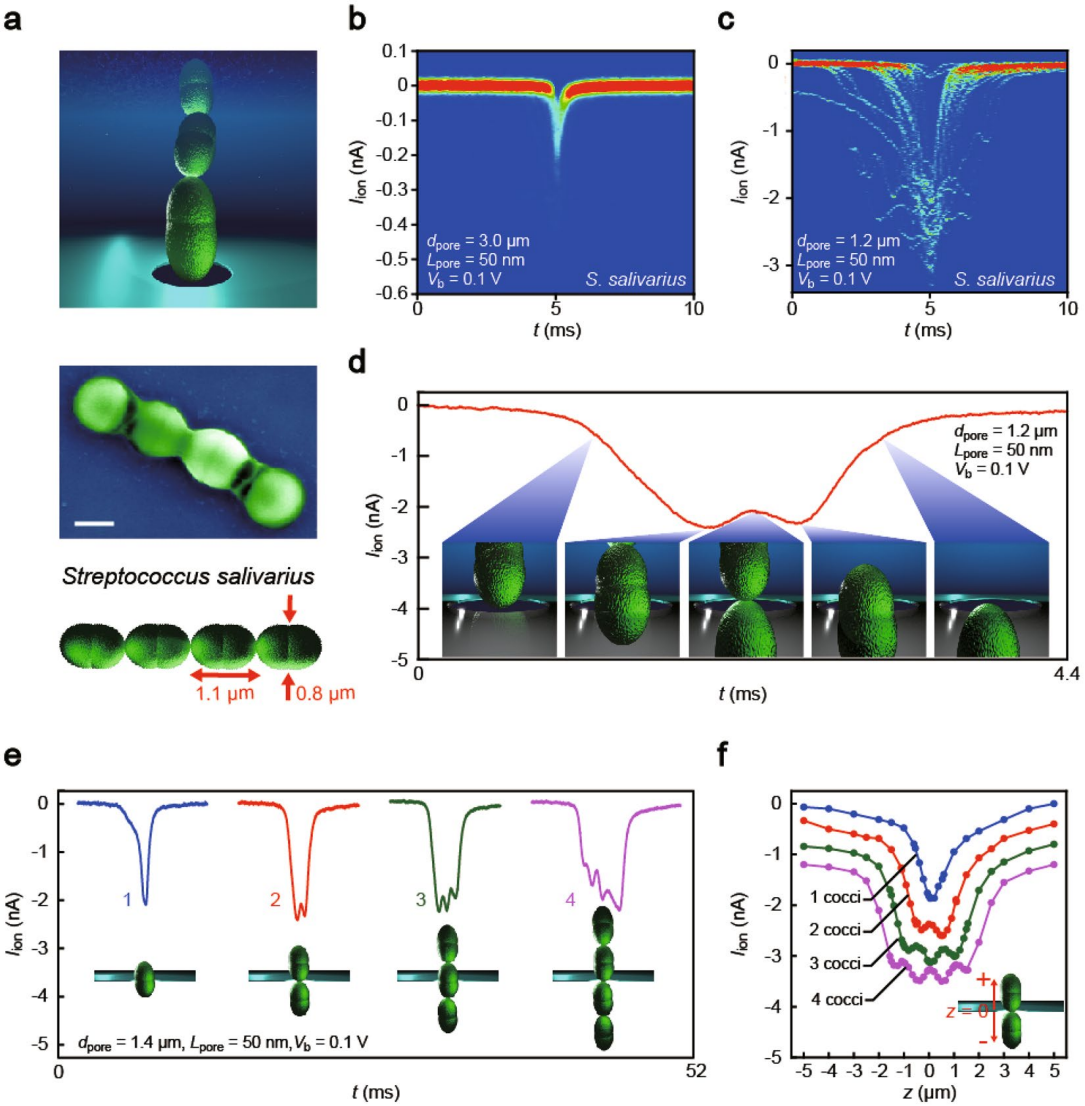
Sensitivity of a low-aspect-ratio pore to particle shapes. (**a**) Schematic and scanning electron microscopy images of *Streptococcus salivarius*. Scale bar denotes 0.5 μm. (**b,c**) Two-dimensional histograms of ionic spike overplots of *S*. *salivarius* in 50 nm thick pores with (**b**) *d*_pore_ = 3.0 μm and (**c**) 1.2 μm. (**d**) Double-peak pulse signal. The *I*_ion_ corrugation depicts the characteristic motif of *S*. *salivarius* as represented in the insets. (**e**) Corrugated spikes showing up to four peaks at the apex. Insets illustrate the bacterial shape deduced from the spike forms. (**f**) Finite element analysis of the ionic current profiles during translocation of the beads-chain-like microbes constructed with one (blue), two (red), three (green), and four (purple) cocci. The curves are shifted vertically for the sake of clarity.

Although the above results suggest the ability of ultra-thin micropores to acquire information concerning morphologies of analytes, it requires more direct evidence to assure the high-spatial resolution of the *microporescopy*. *Streptococcus* is a suitable model system to test the geometrical sensitivity, which consists of micrometer-sized ellipsoidal cells connected in series^27^. We examined a resistive pulse analysis of *Streptococcus salivarius* ^28^ (Fig. 3a), gram positive oral bacteria, using two SiN pores having same depth *L*_pore_ = 40 nm but different diameter *d*_pore_ = 1.4 μm (*AR* ~ 0.03) or 3.0 μm (*AR* ~ 0.01). Whereas the large micropore provided only featureless ionic signatures (Fig. 3b), the bacterium-sized pore yielded peculiar *I*_ion_ spikes with characteristic corrugations (Fig. 3c,d; see also Figs S3–S11). Ionic signatures with up to four bumps were detected in the course of measurement (Figs 3e and S12). Here, each dip is naturally ascribed to translocation of one unit cell (*cocci*) in the bacterial chain through the ultra-thin channel as the ion transport is blocked more (less) effectively at the moment when the cell-to-cell junctions reside in the pore. We performed multi-physics simulations^13^ of the ionic blockade during trafficking of the individual bacteria to verify this by modelling the ball-chain-like bacterial shape as microscale ellipsoidal objects connected in series (Figs 3f and S18–S20). The theoretical *I*_ion_ traces reproduced the characteristic *I*_ion_ oscillations observed experimentally, whereby unequivocally proving the potential of low-*AR* pores for 2D-scanning the nanoscale shape of single particles. Furthermore, the experimental ionic spikes revealed current changes of about 250 pA per translocation of one cocci. The numerical simulations, on the other hand, predicted the corresponding characteristic diameter of each ellipsoidal cell to be 800 nm and 280 nm at the thickest and the narrowest parts, respectively (Fig. S21). The current sensitivity to the radial size of the particles is therefore estimated to be 2 nm/pA. This yields a tentative estimation of the spatial resolution as 26 nm, which is comparable to the pore length of 40 nm, if we only take into account the influence of the rms current noise of 13 pA on a source of error in determining the shape of bacteria (we would like to emphasize that this value is only a tentative one that may change depending on the size and shape of the particles measured).

**Figure 4:**
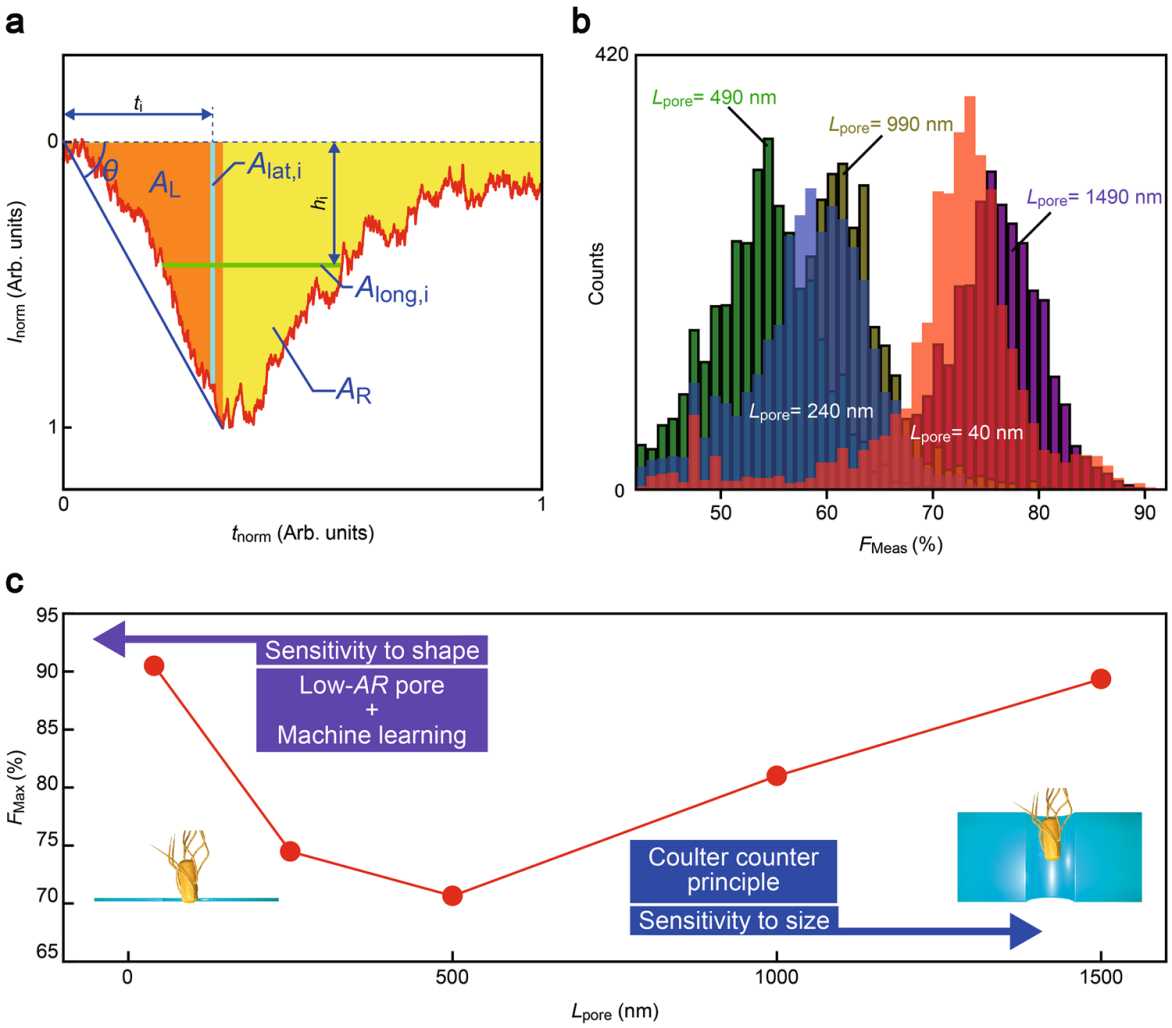
Bacteria discriminations by single-shot pattern analysis. (**a**) Feature parameters characterizing the spike waveforms. *θ* is the angle at the pulse onset. *A*_L_ (orange) and *A*_R_ are the area of the pulse at left and right sides of the peak top, from which the total area *A* = *A*_R_ + *A*_L_ and the ratio *r*_m_ = *A*_L_/*A*_R_ are calculated. In addition, the inertia with respect to the longitudinal (*I*_m_) and transverse (*I*_w_) axes are deduced through ${I}_{{\rm{m}}}=\sum {t}_{i}^{2}{A}_{\mathrm{lat},i}$ and ${I}_{w}=\sum {h}_{i}^{2}{A}_{\mathrm{long},i}$, respectively, where *A*_lat,i_ and *A*_long,i_ are respectively the partial area at *t*_i_ and *h*_i_. (**b**) F-measure score *F*_Meas_ deduced by testing 4020 combinations of feature vectors and classifiers. (**c**) The highest F-measure scores (*F*_Max_) plotted against *L*_pore_. Purple and blue arrows point toward increasing sensor sensitivity to particle shape and size, respectively. While Coulter counter principle predicts an optimal sensitivity to analyte size with a certain pore geometry, lower-aspect-ratio pore channels can benefit from the improved spatial resolution to boost the sensor performance through exploiting the tomographic capability when combined with machine learning based pattern analysis.

Beyond the statistical non-parametric probability density estimation using the artificially-selected features, which has proven effective to assess the number of each bacteria species by using difference in their shape through comparing the sharpness of ionic signatures, we also examined a more general concept of informatics: single-spike identification by machine learning with feature selection based on arbitrary criteria. We employed the Waikato Environment for Knowledge Analysis (WEKA)^29^, a machine learning workbench widely used for data mining, with the Rotation Forest meta-classifier^30^ and forty different algorithms of its base classifiers together with 60 feature parameters including *I*_p_, *t*_d_, and *β*_apex_ (Fig. 4a; see also Supplementary Information 2.2). Intriguingly, the *F*-measure score *F*_Meas_ calculated through a 10-fold cross validation reproduced the *L*_pore_ dependence of the bacterial discriminability (Figs. 4b,c; see Methods for definition of *F*_Meas_), whereby provided further evidence demonstrating the intrinsic sensor capability of low-aspect-ratio pores to sensitively identify minute difference in nanoscale morphologies of individual analytes. Moreover, it is noted that the analytical procedure can be in principle practiced in real-time: off-line learning of the classifiers required as short as 0.003 sec to 2 sec processing times by using a workstation equipped with Intel Core i7 CPU and 32GB RAM, and their on-line single-spike identification spent only 1 μsec to 200 μsec.

**Figure 5:**
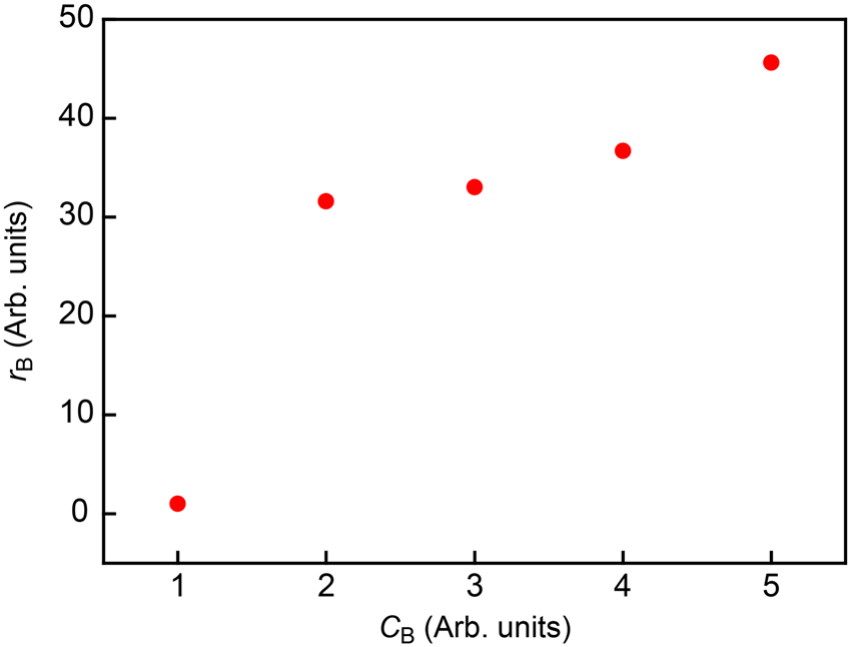
Single-bacteria detections in mixture solution. The relative number of signals judged as *B*. *subtilis r*_B_ with respect to the *E*. *coli* counter part plotted as a function of *B*. *subtilis* versus *E*. *coli* nominal concentration *C*_B_.

From practical viewpoints, it is of importance to evaluate the applicability of the machine learning assisted resistive pulse analyses to a mixture sample of various microbes. For this, we extended the WEKA-based single-bacteria identification to a solution containing *E*. *coli* and *B*. *subtilis* at different relative concentrations using the same teacher data used in the assessments of *F*_Meas_ (Fig. 4). The result revealed a monotonic increase in the relative number of signals assigned as *B*. *subtilis r*_B_ with respect to that of *E*. *coli* with the nominal *B*. *subtilis* to *E*. *coli* concentration ratio *C*_B_ (Fig. 5), thus validating the efficacy of the method to count specific bacteria in a mixture. Meanwhile, the concentration dependence was found to be quite intriguing: A sharp increase in the number of *B*. *subtilis* was observed upon increasing the relative concentration to 2. On the other hand, further increase in *C*_B_ led to non-linear increase in the signal ratio. The non-trivial tendency would reflect the distinct difference in the capture dynamics between the two bioparticles: Compared to *E*. *coli*, which can be drawn into micropores relatively easily whereby giving higher rates of signal occurrence under increased bacteria concentrations, signal detections of *B*. *subtilis* is less efficient presumably due to larger entropic barrier associated with the stiffer cell walls compared to those of *E*. *coli* ^31^.

The present findings have proven the excellent compatibility of sensor informatics to resistive pulse analysis for interfacing ionic current signals and analyte shape in low-aspect-ratio pores. Meanwhile, in light of the sensor performance of existing technologies such as cytometry^32,33^, the bacterial discriminability needs further improvements for practical applications. This would be achieved in part by employing thinner membranes to further enhance the spatial resolution on top of the efforts to bulk test the feasibility for identifying various targets of interest, wherein the emerging two-dimensional nanostructures such as graphene and MoS_2_ are expected to be a key material^34,35^.

## Conclusion

Discriminations of single-bacteria shape was examined by analyzing individual resistive-pulse profiles in low-thickness-to-diameter aspect-ratio pore sensors. Ionic current fingerprints of bacterial morphologies were identified at the signal apex whereat volume-exclusion effects inside the pore by translocating single-bacteria becomes prominent. We also revealed excellent compatibility of a machine learning approach to extract the features relevant to bioparticle shapes in resistive-pulses, which provided data-driven designs of the nanosensor for achieving better discriminability of single-particle shape. Such synergy between physical measurements and machine learning strategies will deliver a versatile strategy to study biology using nanotechnology, wherein biologically-important functions veiled in stochastic noise are identified by extracting relevant parameters from the statistical measurement results by computer in feature space.

## Methods

### Fabrication of low thickness-to-diameter aspect ratio micropores

Solid-state micropore sensors were fabricated as follows. A four-inch Si wafer (0.3 mm thick) whose both sides covered with 50 nm thick SiN layers was first diced into 25 mm × 25 mm chips. Each piece of SiN/Si wafer was then treated with a reactive ion etching with etchant gas of CF_4_ to remove SiN of a small region (0.8 mm × 0.8 mm square) on one side of the surface, wherein a metal sheet was used as a mask. Subsequently, the SiN-removed area was exposed to aqueous solution of KOH at 100 degrees Celsius for deep wet etching of Si. As a result, a 40 nm thick SiN membrane of size 150 μm × 150 μm was formed. Following the process, we delineated a circle pattern on the membrane coated with a resist ZEP520A by an electron beam lithography. After development, we dry-etched the SiN (CF_4_ etchant gas) to drill a pore. Finally, the substrate was immersed in *N*,*N*-dimethylformamide for overnight to remove the remnant resist. For thicker pores, additional SiO_2_ coating was performed on the both sides of the membrane by chemical vapor deposition to increase *L*_pore_ to 240 nm, 490 nm, 990 nm, or 1500 nm.

### Resistive pulse sensing

A micropore chip was sealed with two polydimethylsiloxane (PDMS) blocks from the both sides. On one side of the surface, a microchannel was formed by curing PDMS on an SU-8 mold. The pore substrate as well as the PDMS blocks were exposed to oxygen plasma in prior to the sealing, which served to activate the surface for eternal bonding of PDMS to SiN. After that, three holes were punched in each of the polymer blocks. Two of them were used as inlet and outlet for pouring PBS buffer in the micropore. Bacteria were added only on one side of the pore while the other side was filled only with buffer. The remaining pores were used to insert Ag/AgCl electrodes. A voltage *V*_b_ was applied to these electrodes to electrophoretically drive the bacteria to pass through a pore and record the temporal change in the cross-pore ionic current. A resistance-feedback current amplifier was used to amplify the current and a fast digitizer (NI-5922) backed by a RAID system (NI HDD-8264) was utilized to store the output data at a sampling rate of 1 MHz. All the measurements were carried out at room temperature in air.

### Data analysis

Two dimensional histograms of ionic current spikes were prepared by first extracting the local current minima using a computer program based on Visual Basic 6. Specifically, the open pore current was offset to zero by subtracting the base level through linear fit to 0.5 seconds long *I* − *t* curves cut from the whole data. A threshold of 60 pA was then used to find the current decrease onset in the offsetted ionic traces. Meanwhile, a rising edge at 10 pA was utilized to determine the region wherein a resistive pulse exist. The lowest current values within the peak region was assigned as the peak top of the ionic current spikes. Then, 20 milliseconds of the ionic current data before and after the local minima were collected, thereby obtaining ionic spikes larger than 60 pA. The current versus time two-dimensional histograms were constructed by binning the *I*_ion_ and *t* at 1 pA and 0.1 millisecond, respectively in the Origin Pro software.

### Definition of resistive pulses

An onset of a resistive pulse was searched by referring to the ionic current level at the point where the noise increases to above 5*σ*. *I*_ion_ data were then extracted until the current fluctuations decreased to below 5*σ*. Here, 256 points long margins at both sides of the pulse were also collected. This defines the onset time *t*_s_ for each ionic spike; i.e., 0.256 ms before *I*_ion_ noise rises above 5*σ*. The number of thus obtained ionic spikes were 179 to 557 and differed among the tests (Table S1). For the machine learning analyses, 179 pulses were randomly selected from each set of data.

### Machine learning algorithm

Several features of ionic current spikes were employed for statistical discriminations of bacteria: e.g. the pulse height Ip and width td; the bluntness of resistive pulse apex *β*_apex_ extracted by first normalizing the spike height by *I*_*p*_ as *I*_norm_ = *I*_ion_/*I*_p_ and width by the pulse onset te and the pulse endpoint ts as tnorm = (*t* − *t*_s_)/(*t*_e_ − *t*_s_), and then deduced as ${\beta }_{apex}=1/m{t}_{d}^{2}{\sum }_{i=1}^{m}{({t}_{i}-{t}_{s}-{t}_{ave})}^{2}$ where *t*_*i*_ is the time stamps *I*_ion_ curves intersect the *I*_ion_ level *Y*_th_ % from the peak maxima, *t*_ave_ denotes the arithmetic mean of *t*_i_-*t*_s_ and *m* is the number of intersecting time points; the onset angle *θ* defined by the slope 1/*r* from *I*_norm_ at *t*_e_ to *r*, where *r* is the pulse peak position; the area *A* calculated by dividing a pulse into *n* regions and taking summation of the average *I*_norm_, *h*_i_, at each section; the ratio *r*_m_ between the area at the peak onset and that after the peak maximum; the inertia *I*_m_ and *I*_w_ calculated with respect to longitudinal and transverse axes, respectively (Detailed definitions of each parameter are described in Figs S14–S16). Each parameter (Figs S14 and S15) was coupled to the current vector and the time vector (Fig. S16) to create 60 feature vectors in total. The obtained feature vectors of 161 spikes for each of *E*. *coli* and *B*. *subtilis* (in total 322 spikes) were used as teacher data to judge the other 18 resistive pulses for a test. This procedure is repeated ten times by interchangeably changing the teacher data and the test data within the given 179 spike data, and the total accuracy of each classifier is provided by the average accuracy of the ten tests (ten-fold cross validation method). Data classifications were implemented by using the machine learning workbench WEKA with 67 Rotation Forest ensembles where each used a distinct base classifier. *F*_Meas_ = 2*P*_Pre_ *P*_Rec_/(*P*_Pre_ + *P*_Rec_) was deduced for all of the 60 × 67 = 4040 combinations of the classifiers and the feature vectors, where *P*_Pre_ and *P*_Rec_ are the precision and recall calculated through TP/(TP + FP) and TP/(TP + FN), respectively, with TP, FP, FN being respectively the number of true-positive, false-positive, false-negative outputs. Sufficiently stable accuracies were provided over the ten tests (Supplementary Information 2.3 Statistical variability of the estimation).

## Electronic supplementary material

Supplementary Information
