# Supplementary material for: Discriminating single-bacterial shape using low-aspect-ratio pores
Source: Sci Rep. 2017 Dec 12;7:17371. doi: 10.1038/s41598-017-17443-6 (PMC5727063; doi:10.1038/s41598-017-17443-6)
Supplement: Supplementary file 1 — Supplementary Information [file 41598_2017_17443_MOESM1_ESM.pdf]

Supplementary information for

Discriminating single-bacterial shape using low-aspect-ratio pores

Makusu Tsutsui, Takeshi Yoshida, Kazumichi Yokota, Hirotoshi Yasaki, Takao Yasui, Akihide Arima, Wataru Tonomura, Kazuki Nagashima, Takeshi Yanagida, Noritada Kaji, Masateru Taniguchi, Takashi Washio, Yoshinobu Baba, and Tomoji Kawai

The Supplementary Information includes:

1. Supplementary Figures (Figs. S1-S13)
2. Machine learning approach for ionic spike pattern analysis (Figs. S14-S17)
3. Finite element analysis of ionic current blockage (Figs. S18-S24)
4. Supplementary references

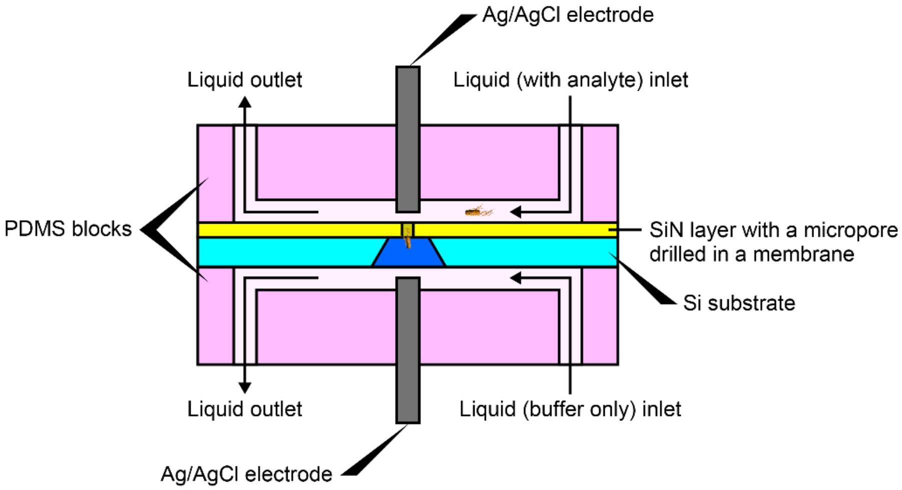

Figure S1. Schematic illustration of a micropore set up. A micropore chip consisting of a microscale hole sculpted in a SiN membrane on a Si substrate is sealed by two polydimethylsiloxane (PDMS) blocks. Two holes in each block serve as inlet and outlet for injecting liquid into the pore. Ag/AgCl electrodes are inserted into another hole for measuring the cross-pore ionic current.

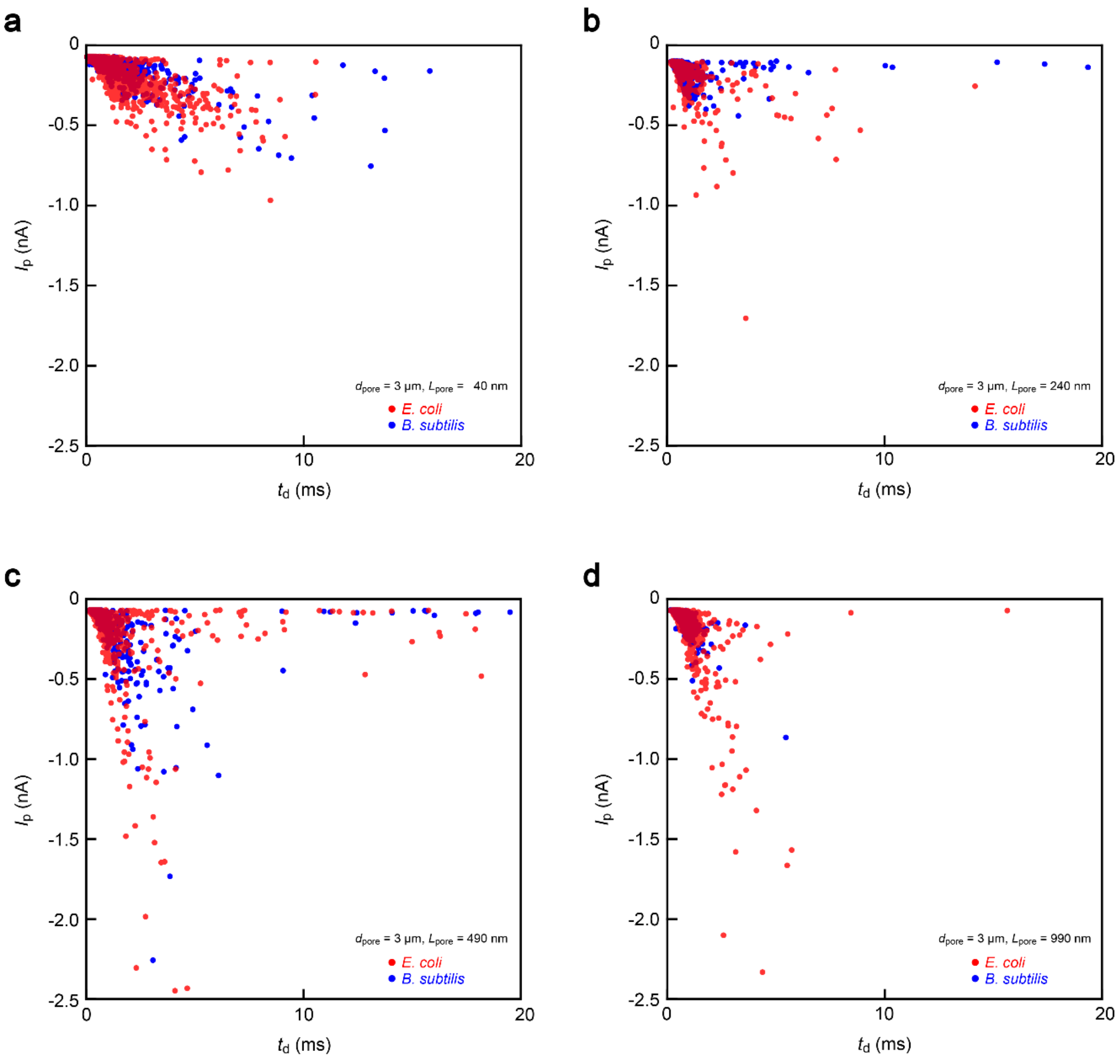

Figure S2. Ionic current spike height I_p versus width t_d scatter plots for *Escherichia coli* and *Bacillus subtilis*. a-d, Data obtained using micropores having diameter and thickness of (a) diameter d_{pore} = 3.0 μm and length L_{pore} = 40 nm; (b) diameter d_{pore} = 3.0 μm and L_{pore} = 240 nm; (c) diameter d_{pore} = 3.0 μm and L_{pore} = 490 nm; and (d) diameter d_{pore} = 3.0 μm and L_{pore} = 990 nm. Red and blue plots correspond to *E. coli* and *B. subtilis*, respectively.

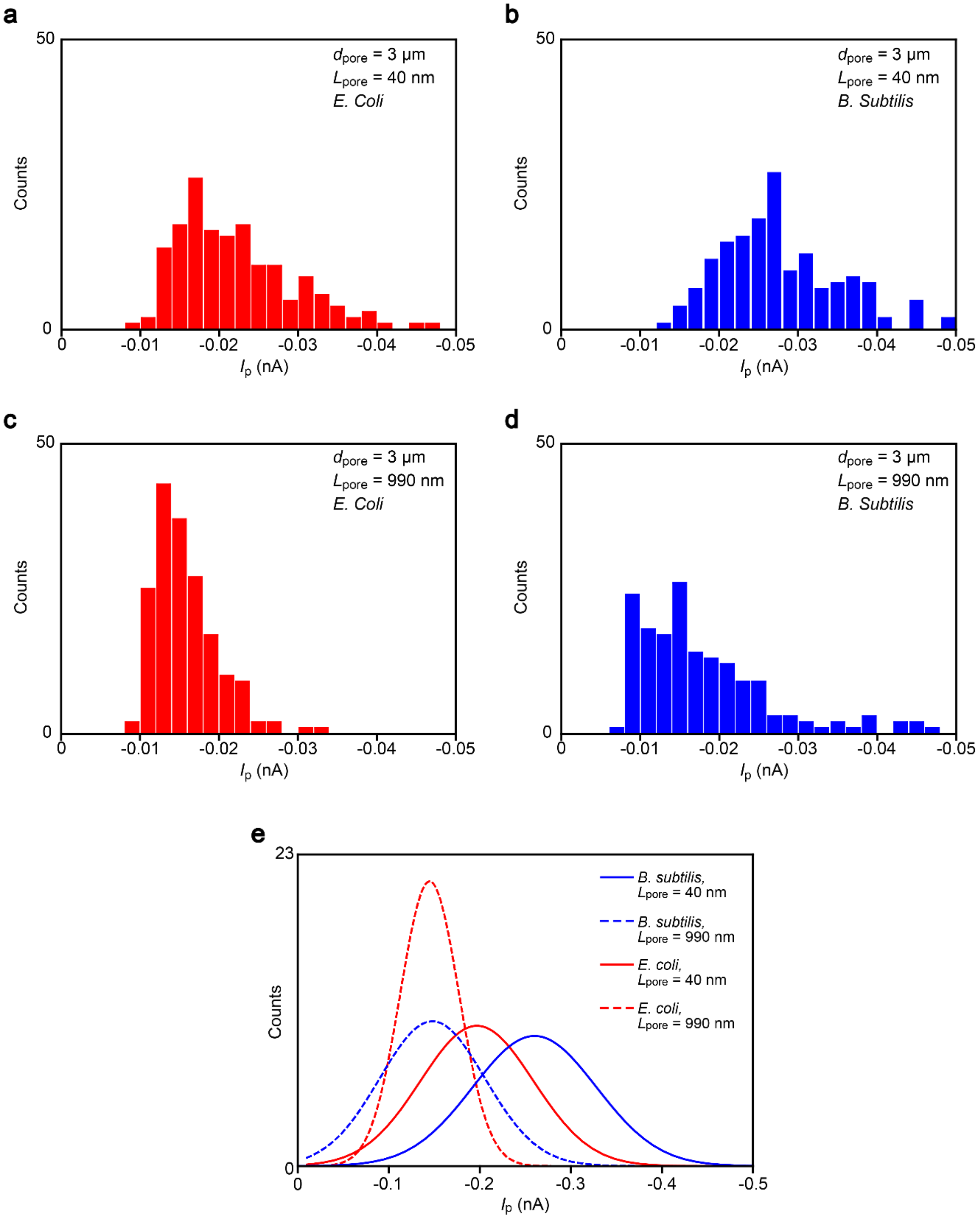

Figure S3. I_p histograms of *E. coli* and *B. subtilis*. a-d, I_p histogram constructed with the ionic current spikes obtained with 3 μm-sized pores with depth L_{pore} = 40 nm (a,b) and 990 nm(c,d). **e,** Gaussian fitting to the I_p distributions. Red and blue lines correspond to the histograms of *E. coli* and *B. subtilis*, respectively.

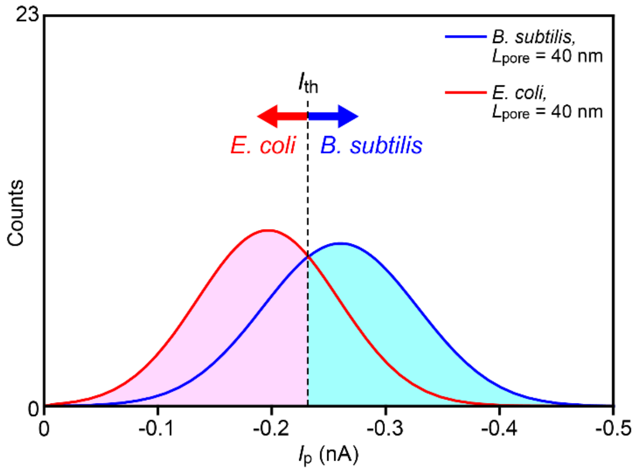

Figure S4. Bacteria discriminability by I_p distributions. Straightforward analysis assigns the ionic spikes with I_p larger than I_{th} as derived from translocation of *B. subtilis* through the micropore whereas those smaller than the threshold to that of *E. coli*. The discriminability deduced from the number of correct reads (summation of the sky-blue and pink area) divided by the whole number of spikes is 69 %.

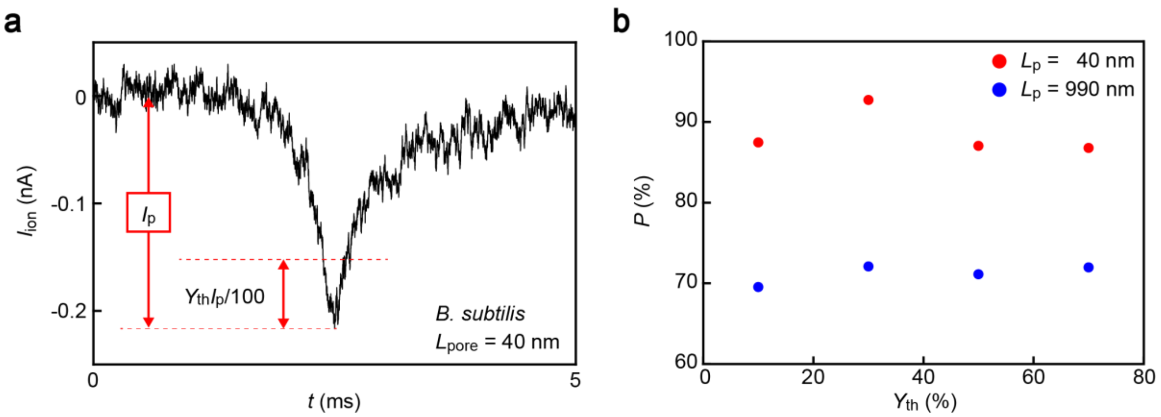

Figure S5. Effects of peak regions on sensor accuracy. a, Bluntness of resistive pulses β_{apex} is given as the deviation of time stamps whereat I_{ion} crosses the current level at $Y_{th}I_p/100$, where I_p is the ionic spike height. Here, Y_{th} in per cent unit defines the amount of peak region used to extract β_{apex}. **b,** Y_{th} dependence of the discriminability *P* of *E. coli* and *B. subtilis* by statistical analysis of β_{apex} and I_p distributions. Results for 3 μm-sized pores with L_{pore} = 40 nm (red) and 990 nm(blue) are shown. *P* changes little with Y_{th} due presumably to the fact that the volume exclusion effect is reflected in almost entire region of the resistive pulses. On the other hand, only limited portions of the ionic current drops are brought by ion exclusion in the low-aspect-ratio pore; in other words, most of the current decrease occurs during the pre-translocation processes where the bioparticles obstruct the ion transport at pore exterior and give rise to appreciable change in the access resistance. This gives rise to the optimal *P* at Y_{th} = 30 % where the particle shape effectively affects I_{ion}. On contrary, as the particle size and shape would matter little on the ionic current blockade outside the pore, the sensor performance is degraded as Y_{th} is increased above 30 %.

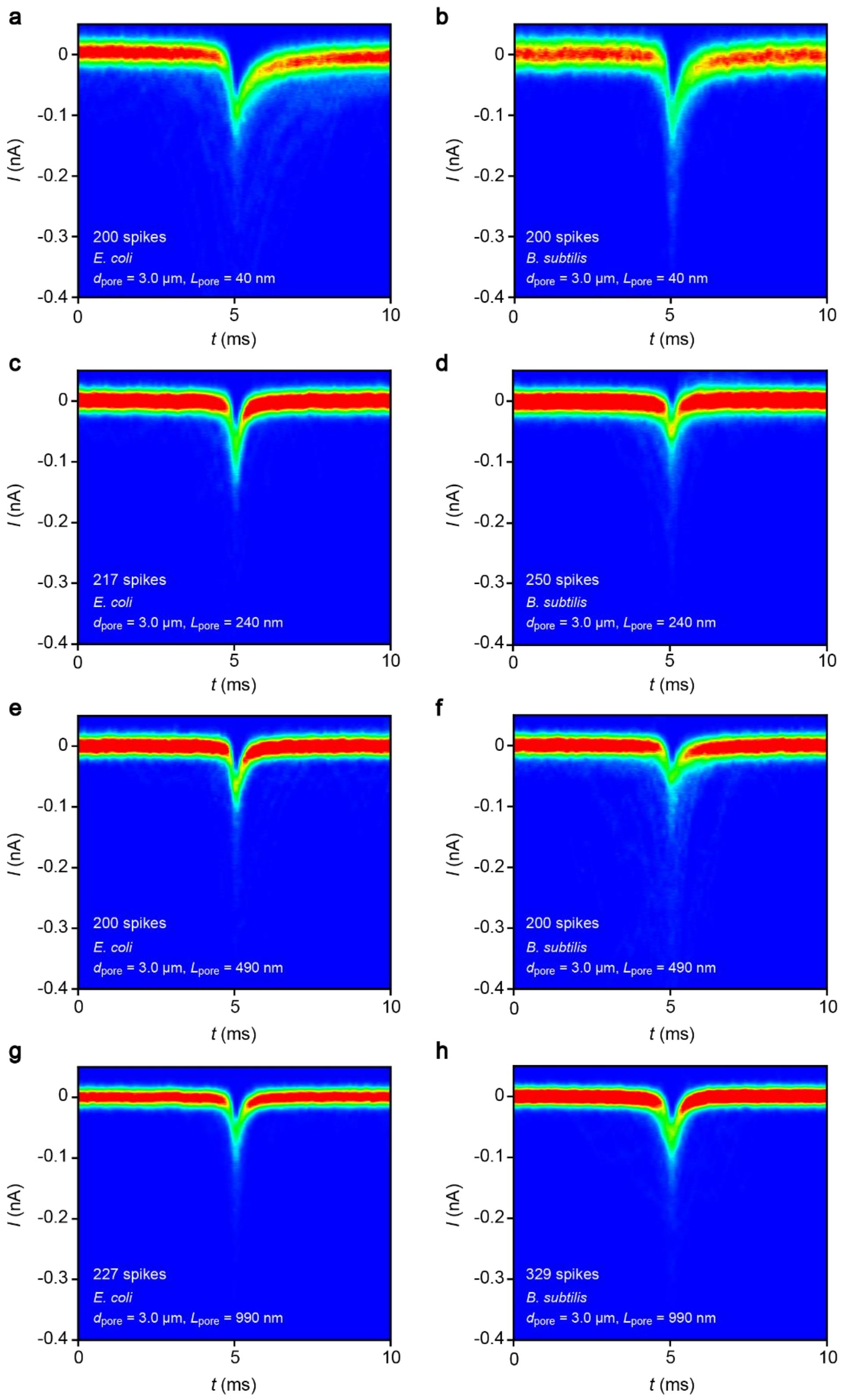

Figure S6. Ionic current spikes obtained for *Escherichia coli* and *Bacillus subtilis* under the applied voltage of 0.05 V using micropore sensors having different

geometries. **a-b,** Two dimensional histograms of resistive pulses obtained with ultrathin micropores of diameter d_{pore} = 3.0 μm and length L_{pore} = 40 nm; **c-d,** d_{pore} = 3.0 μm and L_{pore} = 240 nm; **e-f,** d_{pore} = 3.0 μm and L_{pore} = 490 nm; **g-h,** d_{pore} = 3.0 μm and L_{pore} = 990 nm. Left and right panels correspond to the data for *Escherichia coli* and *Bacillus subtilis*, respectively. Each graph was constructed with data of more than 200 ionic spikes. Base current is offset to zero. The spikes are extracted from the whole traces by finding the resistive pulses and taking out the 20 ms regions before and after the I_{ion} minima.

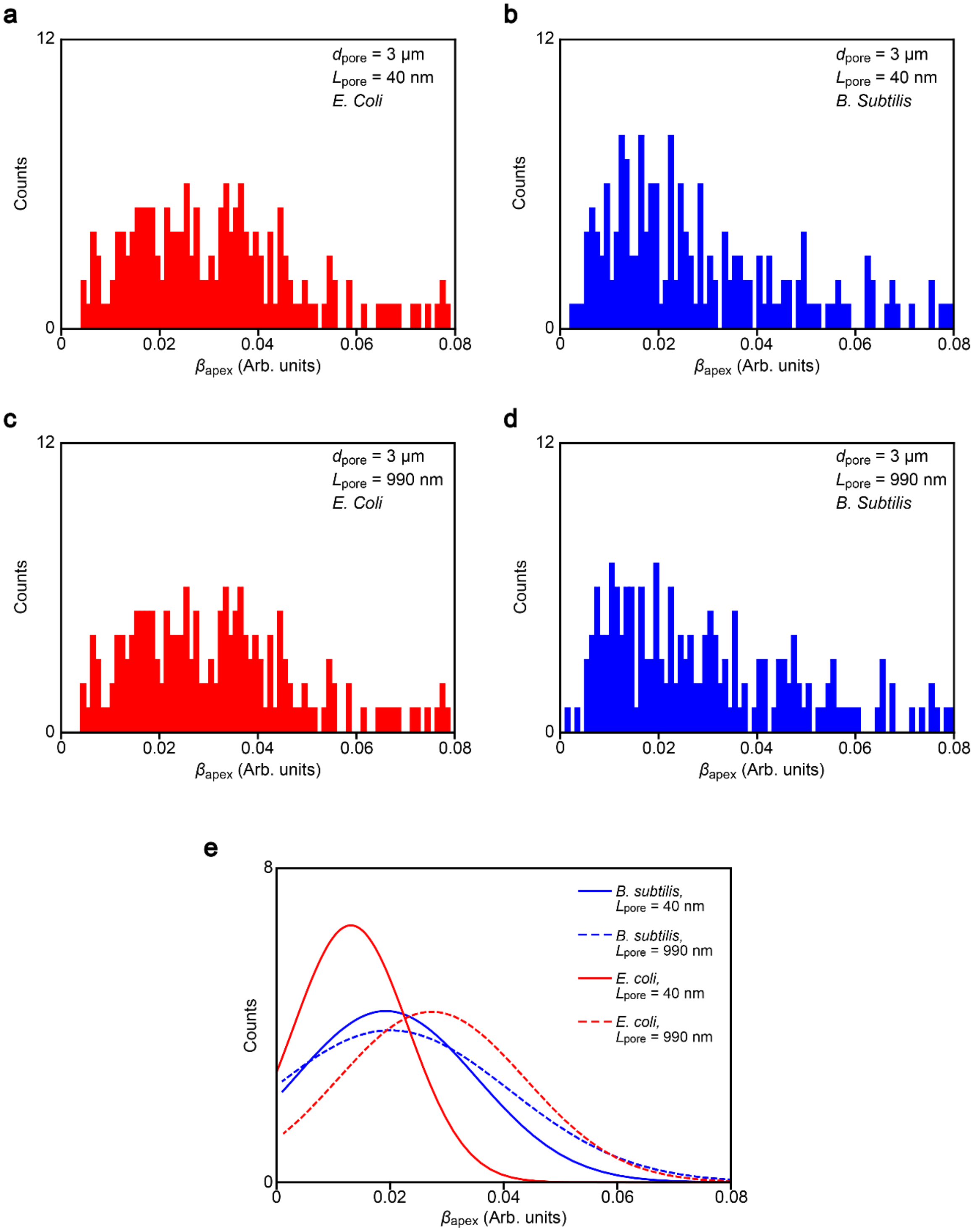

Figure S7. β_{apex} distributions of *Escherichia coli* and *Bacillus subtilis*. a-d, β_{apex} histograms constructed with the ionic current spikes obtained with 3 μm-sized pores with depth L_{pore} = 40 nm (a,b) and 990 nm(c,d). **e,** Gaussian fits to the β_{apex} distributions. Red and blue lines correspond to the histograms of *E. coli* and *B. subtilis*, respectively.

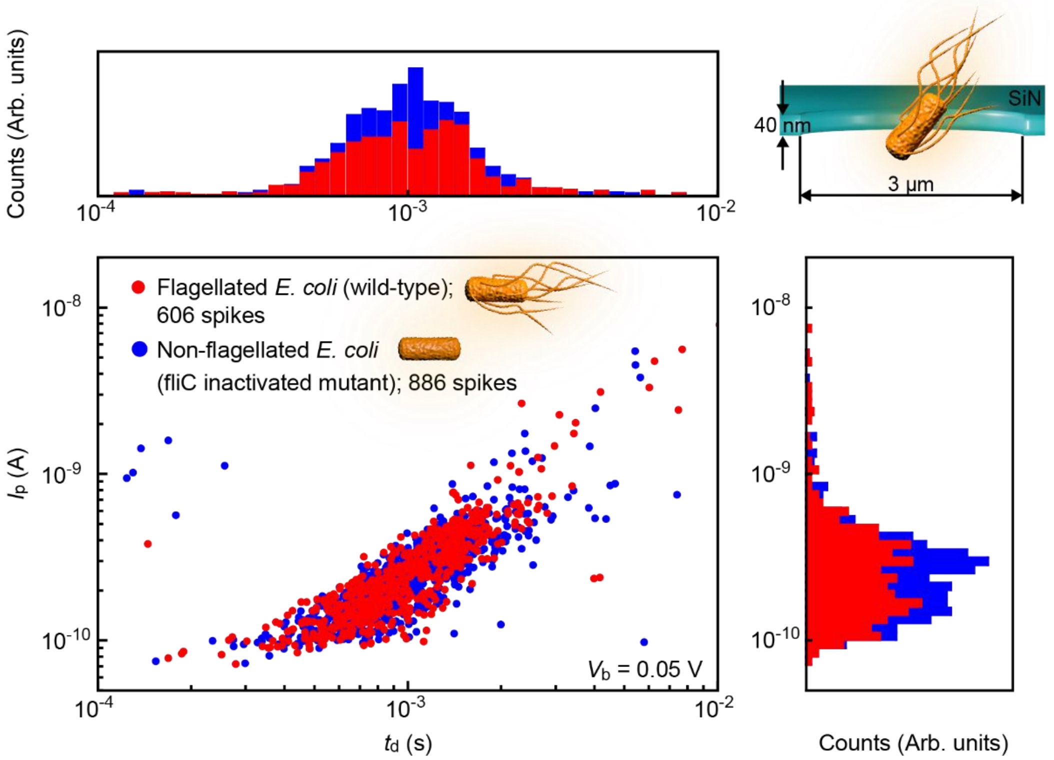

Figure S8. Low-aspect-ratio pore analysis to investigate a role of bacterial motility on the ionic spike line-shapes. More than 500 resistive pulses were obtained for flagellated (wild-type) and non-flagellated (fliC inactivated mutant) *E. coli* in 1/10 diluted PBS buffer under the applied electrophoretic voltage of 0.05 V using 3 μm-sized SiN pore sculpted in a 40 nm thick membrane. The ionic spikes recorded had very similar waveforms as marked by the completely overlapped plots of the pulse height I_p with respect to the width t_d and the corresponding histograms.

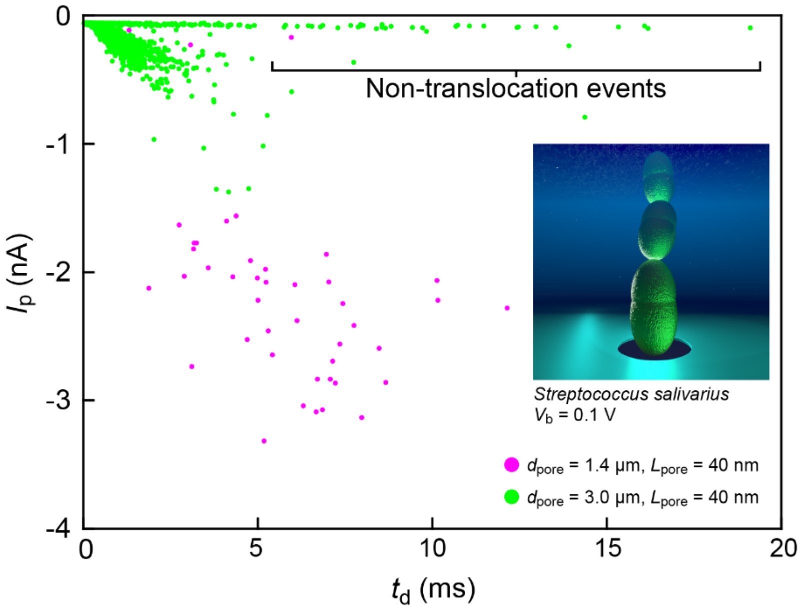

Figure S9. Ionic current spike height I_p versus width t_d scatter plots for *Streptococcus salivarius* obtained using micropores having diameter and thickness of (purple) diameter d_{pore} = 1.4 μm and length L_{pore} = 40 nm and (green) d_{pore} = 3.0 μm and length L_{pore} = 40 nm. The small signals with variable t_d are ascribable to flying-by events of the bacteria skimming through the pore orifice [S1-3].

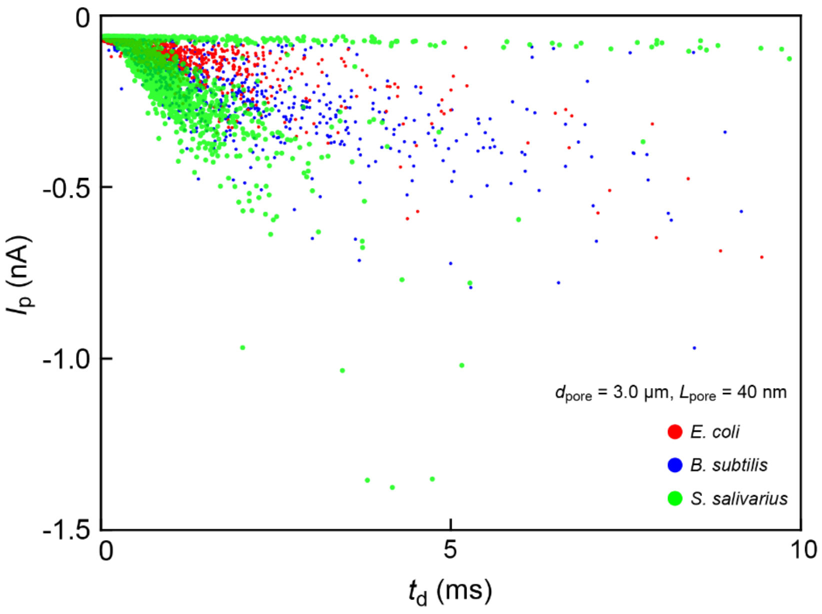

Figure S10. Ionic current spike height I_p versus width t_d scatter plots for three different bacteria, *Escherichia coli* (red), *Bacillus subtilis* (blue), and *Streptococcus salivarius* (green) obtained using micropores having diameter d_{pore} = 3.0 μm and thickness L_{pore} = 40 nm.

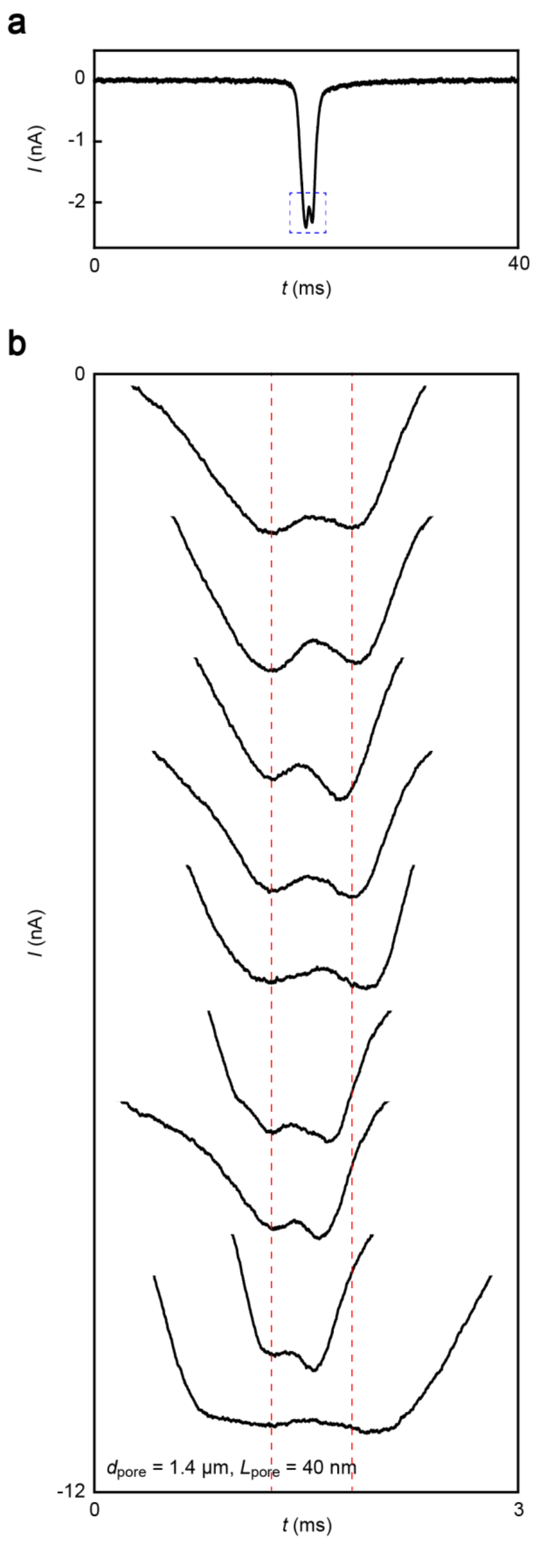

Figure S11. **Ionic current spike wave patterns acquired for *Streptococcus salivarius* with a micropore of d_{pore} = 1.4 μm and thickness L_{pore} = 40 nm showing two-bump feature indicative of translocation of the bacteria having a two-bead**

chain form. a, An example of the corrugated ionic spike. **b,** Magnified views of the peak feature indicated by the square in (a), which display characteristic corrugations reflecting the double-bead shape of the bacteria. Red broken lines are guide to the eyes for the positions of the two minima. The difference in the waveforms are attributed to a variation in the bacterial shape and size and/or the translocation dynamics.

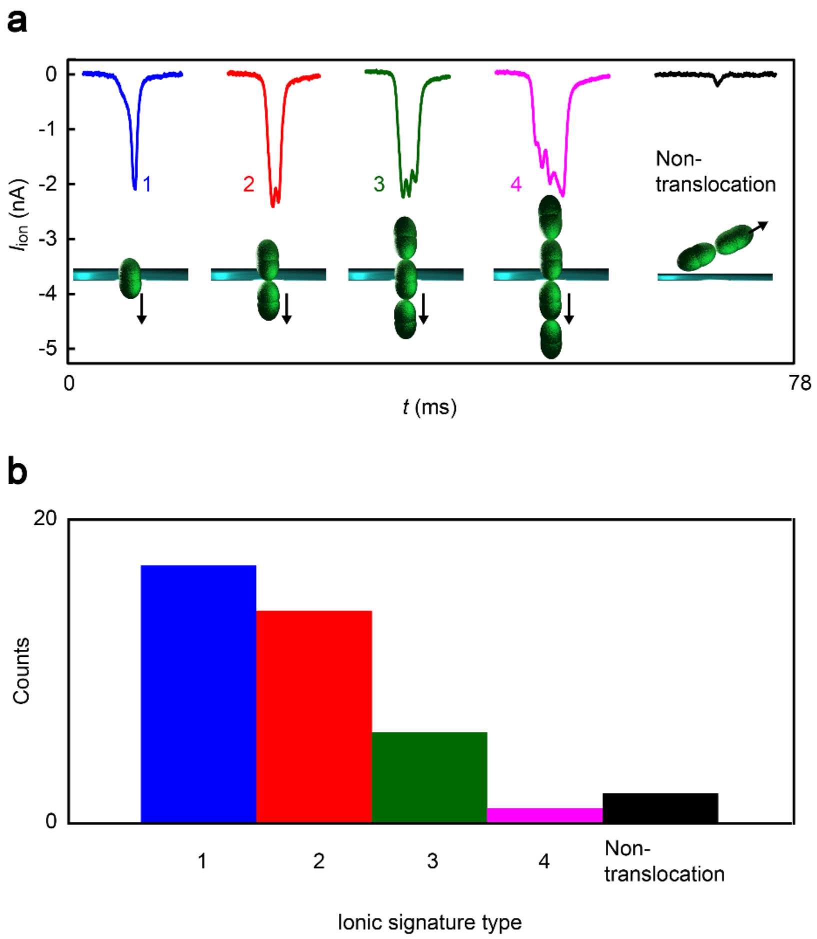

Figure S12. Statistical occurrence of the corrugated ionic spikes of *Streptococcus salivarius* in the microsensor with d_{pore} = 1.4 μm and thickness L_{pore} = 40 nm. a, Five distinct types of the ionic spike wave patterns: A peak with single (1, blue), double (2, red), triple (3, green), or quadruple (4, purple) bump feature; and significantly weak pulses (black). **b,** The number of each type of spikes detected.

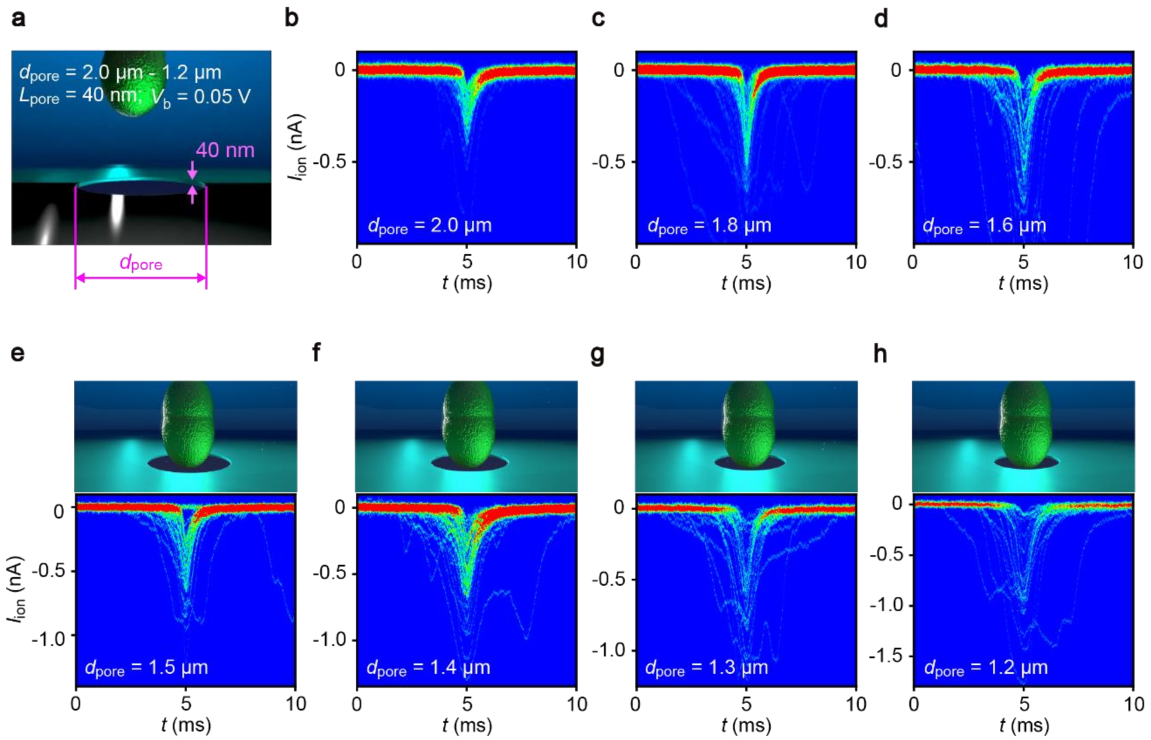

Figure S13. Dependence of the ionic current spike line-shapes of *Streptococcus salivarius* on the pore diameter. a, Micropores of different diameter d_{pore} ranging from 2.0 µm to 1.2 µm and the same length of L_{pore} = 40 nm were used to record resistive pulses during translocation of single *S. salivarius* in 1/10 diluted PBS buffer under the applied dc voltage V_b of 0.05 V. **b-h,** Two dimensional histograms of resistive pulses obtained with the ultrathin micropores of (b) d_{pore} = 2.0 µm, (c) 1.8 µm (d) 1.6 µm, (e) 1.5 µm, (f) 1.4 µm, (g) 1.3 µm, and (h) 1.2 µm. Only single-peak pulses were obtained for pores of larger than 1.6 µm. On the other hand, the characteristic corrugations in ionic spikes reflecting the bead-chain structure of *S. salivarius* were observed in pores smaller than 1.5 µm.

2. Machine learning approach for ionic spike pattern analysis

2.1 Statistical estimations of the respective numbers of *E. coli* and *B. subtilis*

Statistical estimations of the respective numbers of *E. coli* and *B. subtilis* were conducted by applying non-parametric probability density estimation of each bacteria in feature spaces where every feature space is formed by a pairwise combination of distinct features of the ionic current waveforms. Specifically, we chose the pulse height I_p and width t_d (Fig. S14a), the bluntness β_{apex} of the apex (Fig. S14b), the onset angle θ (Fig. S12a), the area A (Fig. S15b) and its ratio r_m before and after the spike maxima (Fig. S15c), and the momenta with respect to the longitudinal (I_m; Fig. S15d) and transverse axes (I_w; Fig. S15e). Once the distribution of the pairs of the pulses in the feature space is given, the distribution is approximated by a weighted combination of the non-parametric probability densities learnt beforehand for the two bacteria species. The weights which provide optimal approximation are proportional to the respective numbers of the two bacteria species.

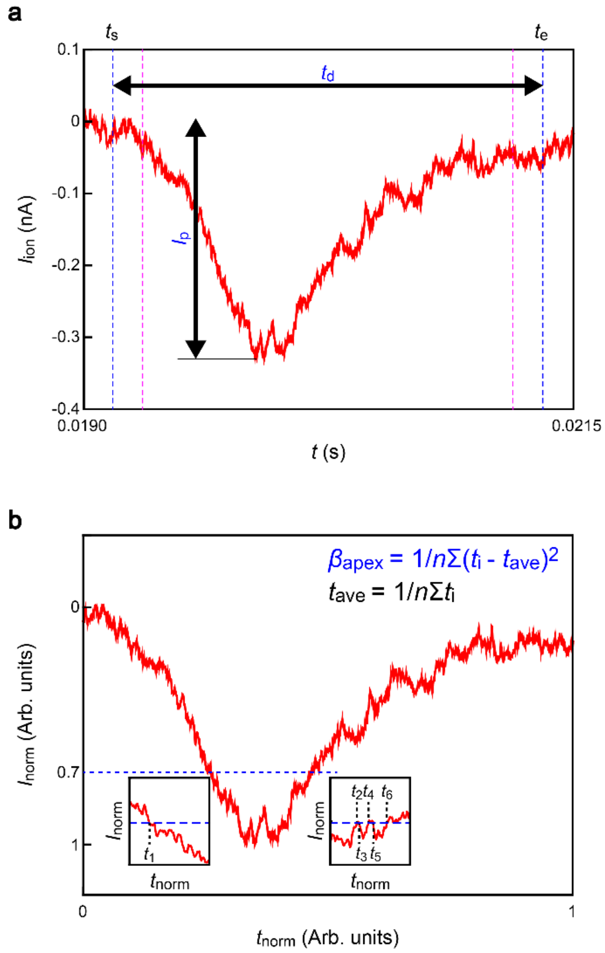

Figure S14. Peak features: pulse height, width, and bluntness. **a,** A resistive pulse was extracted by using 5σ as a threshold to find a pulse onset. The time whereat the

fluctuations of the ionic current I_{ion} decreases below 5σ was also identified (red lines). Furthermore, the peak regions were expanded by 0.256 ms at both sides (blue lines) whereby defining the regions of ionic current spikes. In the figure, t_s and t_e denote the onset and the end position of the resistive pulse, respectively. The pulse width $t_d = t_e - t_s$ and the pulse height I_p are the feature parameters used for pattern analysis. **b,** The bluntness of resistive pulse apex β_{apex} was extracted by first normalizing the spike height by the height I_p as $I_{norm} = I_{ion} / I_p$ and width by t_e and t_s as $t_{norm} = (t - t_s) / (t_e - t_s)$. Then, β_{apex} was deduced as $\beta_{apex} = 1/m{t_d}^2 \sum_{i=1}^{m}(t_i - t_s - t_{ave})^2$ where t_i is the time stamps whereat I_{ion} curves intersect the I_{ion} level Y_{th} % from the peak maxima. In the description, t_{ave} and m denote the arithmetic mean of t_i-t_s and the number of intersecting time points, respectively.

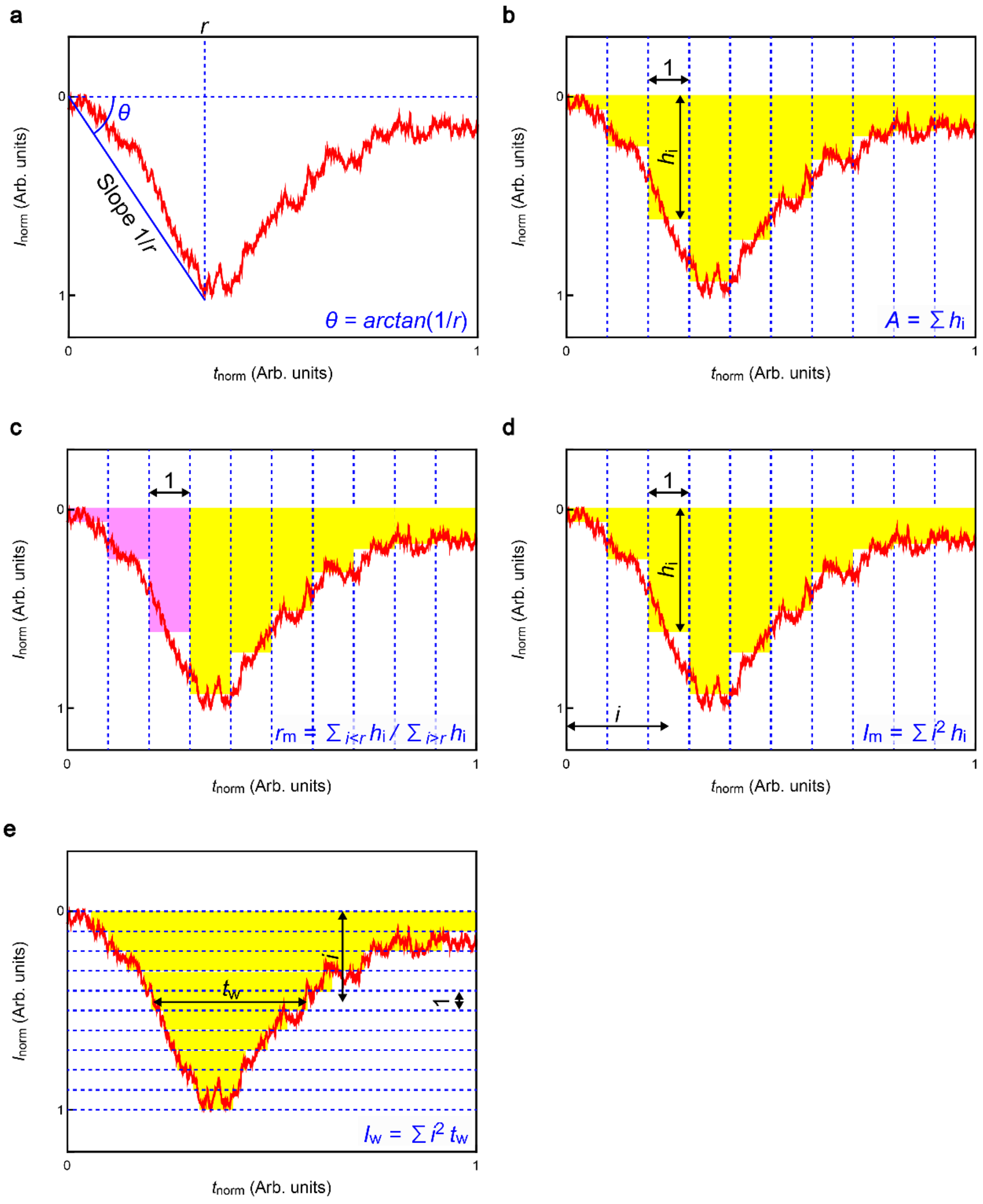

Figure S15. Peak features: onset angle, pulse area and its ratio, and longitudinal and transverse momenta. **a,** The onset angle θ defined by the slope $1/r$ from I_{norm} at t_{norm} = 0 to r, where r is the peak position. **b,** The area A is calculated by dividing a pulse into n regions (dotted lines) and taking summation of the average I_{norm}, h_i, at each section. **c,** The ratio r_m between the area at the peak onset (purple) and that after the peak

maximum (yellow). **d-e,** The inertia I_m (d) and I_w (e) calculated with respect to longitudinal and transverse axes, respectively.

2.2 Single-bacteria discriminations

Single-bacteria discriminations were performed by pattern-analyze individual ionic current spikes using classification algorithms of machine learning. For this, we also used the aforementioned distinct features of the ionic current pulse waveforms. In addition to these parameters, the line shapes of resistive pulses were characterized in more general sense by the vectors in the current (Fig. S16a) and time domains (Fig. S16b):

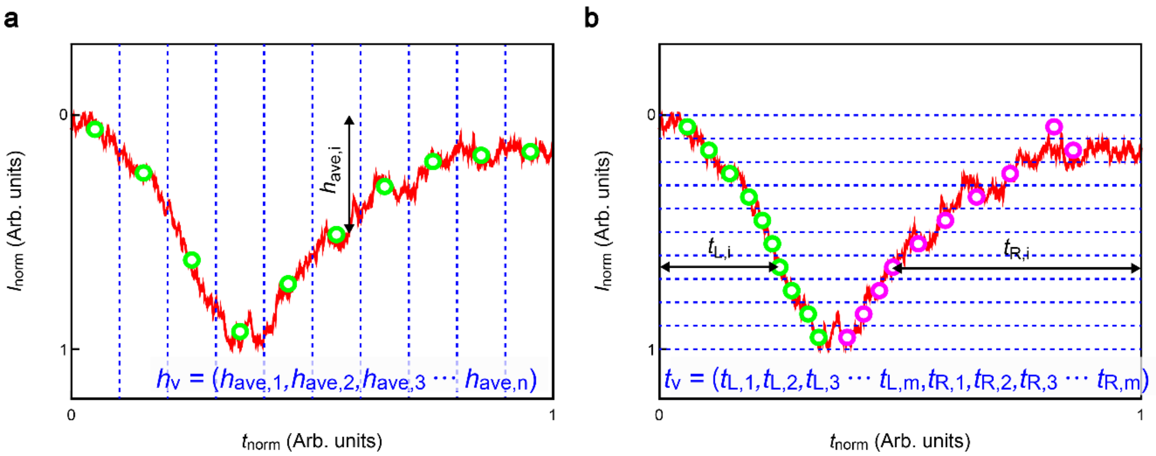

Figure S16. Peak features: current and time vectors. **a,** Current vector h_v that consists of a sequence of the average I_{norm}, $h_{ave,i}$ (green circles), at ith section from i = 1 to n. Five different vectors were obtained by changing the division number n as 8, 16, 32, 64, or 128. **b,** Time vector t_v defined as a sequence of the average time $t_{L,i}$ and $t_{R,i}$, at ith section from i = 1 to m, where L and R denote the regions before and after the pulse peak (m = 8, 16, 32, 64,or 128).

We created 60 different feature vectors by combining the above mentioned parameters as follows:

Combinations of feature parameters	Number of vectors
1. h_v (n = 8, 16, 32, 64, 128)	5
2. h_v + (I_p,t_d, β_{apex}, θ, A, r_m, I_m, I_w)	5
3. h_v + non-normarized t_v (n = 8, 16, 32, 64, 128; m = 8, 16, 32, 64, 128)	25
4. h_v+ normarized t_v (n = 8, 16, 32, 64, 128; m = 8, 16, 32, 64, 128)	25

Pattern analysis of ionic current spikes of *E. coli* or *B. subtilis* was exhibited on basis of the thus acquired dataset using The Waikato Environment for Knowledge Analysis (Weka) machine learning workbench with 67 Rotation Forest ensembles where each applies a distinct base classifier such as naïve Bayes models. In this supervised learning, 161 pulses of each bacteria, either *E. coli* or *B. subtilis*, (in total 322 vectors) was used as teacher data to judge the other 18 spikes (While up to 500 pulses were obtained in some measurements (see Tab. S1), we randomly selected 179 pulse data from the larger pools to keep the number of pulses the same for the machine learning analysis). The output was further evaluated to deduce F-Measure score $F_{\mathrm{Meas}} = 2P_{\mathrm{pre}}P_{\mathrm{Rec}}/(P_{\mathrm{Pre}} + P_{\mathrm{Rec}})$, where P_{Pre} and P_{Rec} are the precision and recall calculated through TP/(TP + FP) and TP/(TP + FN), respectively, with TP, FP, FN denoting respectively the number of true-positive, false-positive, false-negative cases.

2.3 Statistical variability of the estimation

We used the ten-fold cross validation which is standard in statistics and machine learning for evaluating the accuracy of the classifiers. In this scheme, the experimentally obtained data set consisting of the spikes and their types such as *E. coli* and *B. subtilis* is partitioned into ten blocks containing an equal number of spikes. Then, in a fold stage, the spike data with the bacteria types in the nine blocks among the ten are used for the classifier learning, and the accuracy of the classifier is evaluated by matching the estimated types and the experimentally known types of the individual spikes in the rest test block which have not been seen by the classifier yet. This fold stage is repeated ten times by interchangeably changing the nine learning blocks and the rest test block, and the total accuracy of the classifier is provided by the average accuracy of the ten tests. The idea behind this 10-fold cross validation scheme is to properly evaluate the accuracy of the classification method for unseen spikes while ensuring less experimental variability of the data and better efficiency to use the limited amount of the data.

In our experiment, the variability of the classification accuracy over the ten tests remains 4-8% in terms of the standard deviation (Fig. S17). Since the identifications of the individual spikes by the classifiers should be mutually independent and have an identical error risk when the experimental condition was well maintained, this resultant accuracy

should follow a binominal distribution of true and false identifications. Thus, the standard deviation of the accuracy is expected to be $\sqrt{P_{Pre}(1-P_{Pre})/\mathrm{N}} \cong \sqrt{0.8(1-0.8)/36} = 6.7\%$ where N is the number of the spikes in the test data. Accordingly, the variability of the accuracy demonstrated in our experiment is very reasonable to conclude that the experiment was statistically well controlled during the data acquisition.

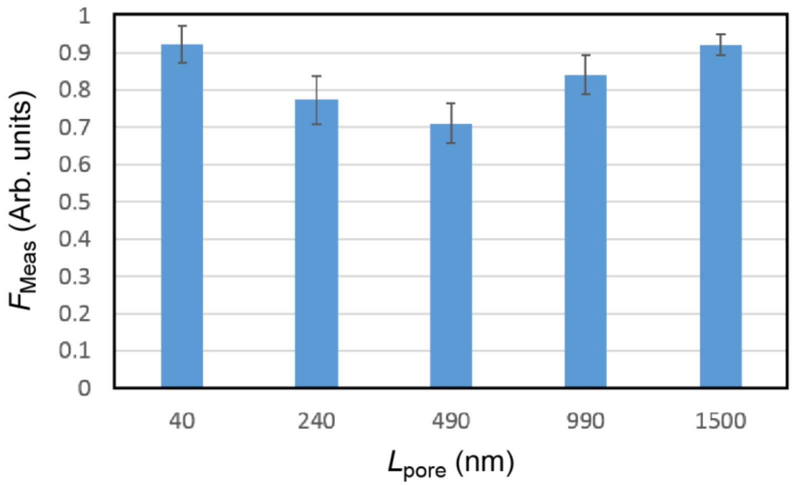

Figure S17. The maximum F-measures and their standard errors for 5 thicknesses of the pores. The maximum F-measures achieved by the forty base classifiers with the Rotation Forest meta-classifier are depicted for the pores having the 5 thicknesses. The F-measure and its error bar for each thickness are the average F-measure and its standard deviation across the ten-fold cross validation on the pore.

Table S1. The number of resistive pulses recorded.

L_{pore} (nm)	*Bacteria*	*# pulses*
40	*E. coli*	179
40	*B. subtilis*	480
240	*E. coli*	388
240	*B. subtilis*	288
490	*E. coli*	343
490	*B. subtilis*	299
990	*E. coli*	557
990	*B. subtilis*	447
1500	*E. coli*	194
1500	*B. subtilis*	442

3. Finite element analysis of ionic current blockage

3.1 Physical model for finite element analysis.

To simulate the electric current density j at a steady state, we use the convergence condition expressed as $\nabla j = 0$. The ionic current through a nanopore is given by

$$\nabla j = -\nabla[(\sigma_{\mathrm{W}} + F\sum_{\mathrm{i}} z_{\mathrm{i}}^2 u_{\mathrm{i}} c_{\mathrm{i}})\nabla V_{\mathrm{c}} + F\sum_{\mathrm{i}}(z_{\mathrm{i}}^2 D_{\mathrm{i}} \nabla c_{\mathrm{i}} - z_{\mathrm{i}} c_{\mathrm{i}} U)] = 0 \qquad 1.$$

Here, V_c, σ_w and F are the electric potential, the electrical conductivity of water (5.5×10^{-6} S/m) and the Faraday constant (9.649×10^{4} C/mol), respectively, while z_i, u_i, and c_i denote the charge, the electrical mobility and the concentration for ion species of i. In this study, as we used 10 times diluted phosphate buffered saline (0.1×PBS) as the electrolyte, we approximated it by NaCl 13.7 mmol/L in this simulation. We used $z_i = 1$, u_i, = 5.194×10^{-8} m^2/Vs for i = Na, and $z_i = -1$, u_i, = 7.912×10^{-8} m^2/Vs for i = Cl. Contributions from diffusion and convection are included in the expression as $-F\sum_{\mathrm{i}} z_{\mathrm{i}}^2 D_{\mathrm{i}} \nabla c_{\mathrm{i}}$ and $F\sum_{\mathrm{i}} z_{\mathrm{i}} c_{\mathrm{i}} U$, respectively, where D_i is diffusion constant for ion species i expressed as $u_i k_B T$ (k_B and T are the Boltzmann constant of 1.381×10^{-23} J/K and temperature. We used T = 293.15 K in this simulation.) by the Einstein relation, and U is the velocity of electrolyte solution.

Meanwhile, not only current flow but also static charge plays a role in the electric current in nanopore devices, because it describes the spatial distribution of ions (c_i) in the electric double layer on solid–electrolyte interfaces. The distribution of ions at the equilibrium state is given by Boltzmann statics $c_i = c_i^0 \exp(-z_i e V_s / k_B T)$, where c_i^0, e, and V_s denote the ion concentration for i ion at the zero potential, the elementary charge, and electrostatic potential. Hence, the Poisson–Boltzmann equation is described by

$$\nabla^2 V_{\mathrm{s}} = -\frac{\rho}{\varepsilon_{\mathrm{w}}} = -\frac{1}{\varepsilon_{\mathrm{w}}} F\sum_{\mathrm{i}} z_{\mathrm{i}} c_{\mathrm{i}} \exp(-z_{\mathrm{i}} e V_{\mathrm{s}} / k_{\mathrm{B}} T) \qquad 2.$$

Here, ρ and ε_w are effective static charge by ions and the permittivity of water (ε_w = 7.083×10^{-10} F/m). Also, Eq. 2 was simultaneously solved with Nernst–Plank equation given by

$$\nabla J_i = \nabla(-D_i \nabla c_i - z_i \mu_i F c_i \nabla V_s) + U \nabla c_i = 0$$

3,

where, J_i is the ion flux for i ion. The first, second, and third terms denote diffusion, electrical migration, and convention, respectively.

The velocity of electrolyte solution *U* in Eq. 1 and 3 was calculated by incompressible Navier–Stokes equation expressed as

$$-\nabla p + \mu \nabla^2 U + f = 0 \qquad 4,$$

where *p* and *μ* are pressure and dynamic viscosity of water (1.009×10^{-3} Ns/m^2). *f* is the body force applied to electrolyte solution, and we defined it as $f = -\rho \nabla V_c$ by assuming the electric dragging force for electrolyte solution. All calculations were performed by COMOL Multiphysics 5.0.

3.2 Structural model and boundary condition for finite element method (FEM).

We used the model of two-dimensional space together with a rotational symmetry to simulate the straight translocation of bacteria via naopore, which possess a cylindrical configuration. The structure of *S. salivarius* was modeled in reference to the scanning electron microscope image (Fig. S18), and four type models, in which each *S. salivarius* possess 1-4 cocci, were created to simulate experimentally-observed current peak signatures.

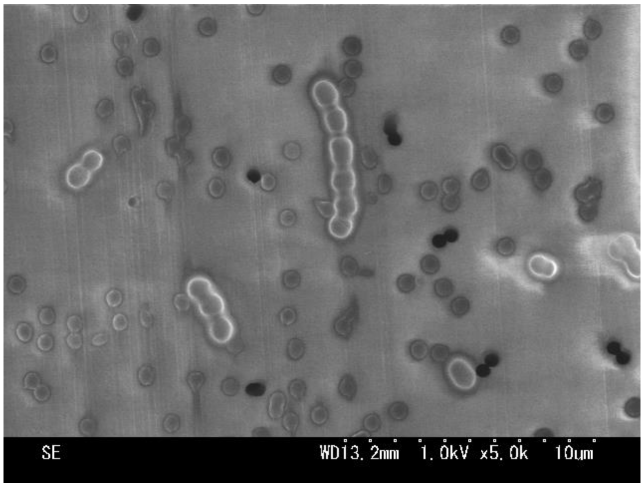

Figure S18. A SEM image of *S. salivarius*. Small and dark circular images are pores of filters used to place only the bacteria on the substrate.

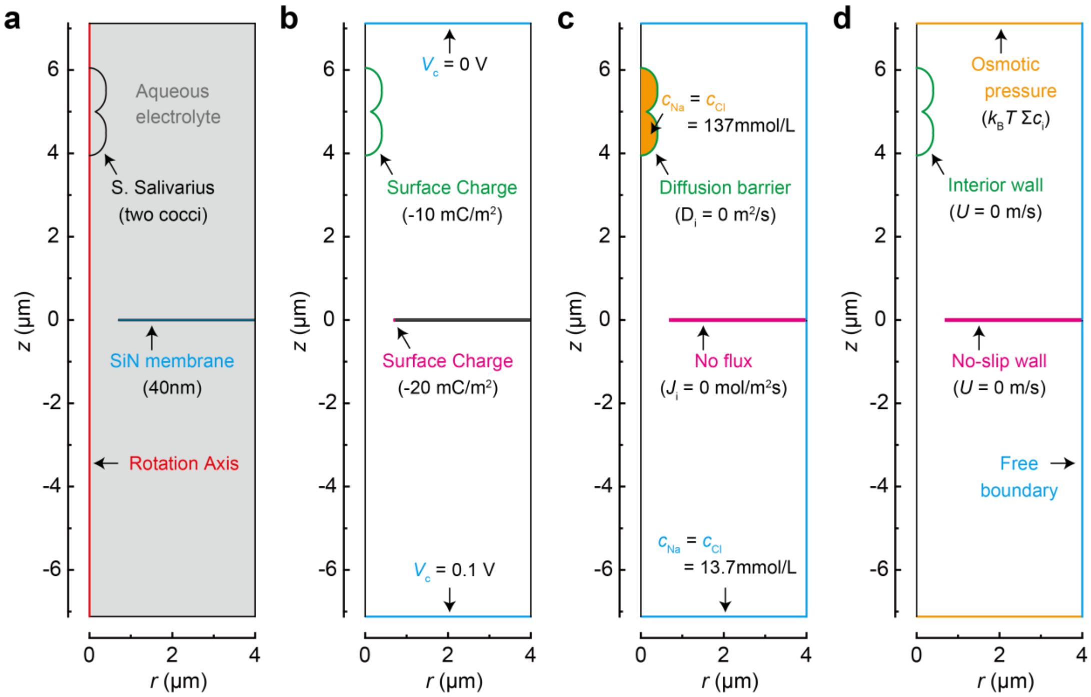

Figure S19. Schematic illustrations for the constructed model for the investigated system. As an example, *S. salivarius* of two cocci located at z = 5 µm is shown. **a,** The conditions for geometry and materials, **b,** electricity, **c,** ion transport, and **d,** liquid flow are exhibited.

The model for two cocci *S. salivarius* is depicted in Fig. S19, where a case at z position of 5 μm is provided. The model for the nanopore device was constructed as follows. The radius and height for *cis*- and *trans*- chambers were 4 μm and 7.1 μm, respectively. A 40 nm thick SiN membrane (a conductivity of 1.0×10^{-14} S/m and a permittivity of 1.851×10^{-11} F/m were used for SiN) with a 0.7 μm radius pore is placed between the *cis*- and *trans*- chambers as seen in Fig. S20a. The simulation for the translocation of *S. salivarius* via the nanopore was conducted by sweeping the center position of *S. salivarius* from z = 5 μm to z = −5 μm.

Subsequently, we set up boundary conditions for solving the differential equations 1–4. Figure S19b exhibits the boundary condition for the dependent variables of V_c and V_s. The top side corresponds to the ground, and a bias voltage of 0.1 V is applied to the bottom terminal side. The total current through the nanopore was computed by a surface integral on this terminal according to Eq. 1. A surface charge of −10 mC/m^2 at the *S. salivarius* was defined by taking account of a zeta potential of −18.6 mV measured by means of dynamic light scattering (DLS). Figures S19b and c depict the conditions for Eqs. 3 and 4 of which dependent variables are c_{Na}, c_{Cl}, p and U. Inside the *S. salivarius*, as the ion concentration would be assumed to 1×PBS, we set $c_{Na} = c_{Cl}$ = 137 mmol/L. The surrounding boundaries were $c_{Na} = c_{Cl}$ = 13.7 mmol/L based on the experimental condition of 0.1×PBS for electrolyte, and we assumed these boundaries were allowed to exchange ions and energy with an external system. Contrast to these boundaries, the sold–liquid interfaces such as SiN–electrolyte and *S. salivarius*–electrolyte are forbidden to exchange ions and energy (J_i = 0 mol/m^{2}s, D_i = 0 m^2/s, and U = 0 m/s).

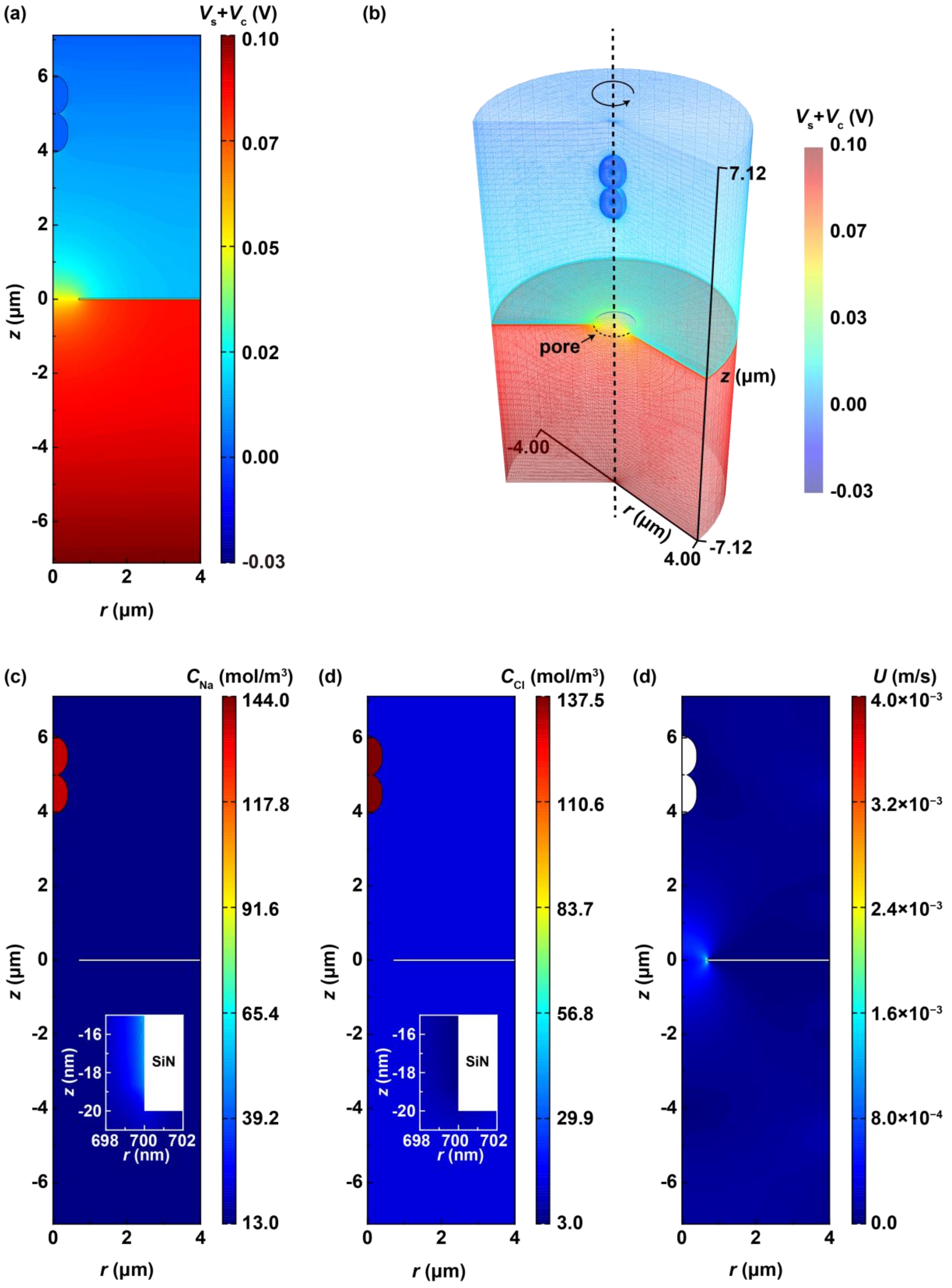

Figure S20. Simulated results of the invariables of **a, b,** V_s+V_c, **c,** c_{Na}, **d,** c_{Cl} and **e,** *U* for the model illustrated in Fig. S19. As the constructed model has rotational axis at *r* = 0, the practical 3D view is given by the revolution, seen in the relation between **a** and **b**. Insets in **c** and **d** are the enlarged view at the interface between the surface of SiN for the pore and electrolyte.

The prepared models were meshed by extremely fine condition and the corners and layer boundaries of those were refined enough to simulate the physics within Debye-length of $\lambda_D = \sqrt{\frac{\varepsilon_W k_B T}{N_A e^2 c_i}}$, where N_A is Avogadro constant of 6.02×10^{-23} mol^{-1}. On the condition in this study, $\lambda_D \approx 3.5$ nm is evaluated. As an example of the simulated results, the independent variables of $V_s + V_c$, c_{Na}, c_{Cl}, and U are depicted in Fig. S20, of which model corresponds to that of Fig. S19. V_s and V_c respectively represent the electric potential solved by Eq. (1) and (2), that is, $V_s + V_c$ illustrates the total electric potential in the given model as seen in Fig. S20a. Because of the presence of the rotational symmetry, the physical properties for the pore devices are eventually given by the revolution of the prepared models as shown in a cylindrical shape of Fig. S20b. Figs. S20c and d indicates the distribution of c_{Na} and c_{Cl}, and also each inset figure is for that of the interface between the inner wall of the pore and electrolyte liquid. Due to the negative surface charge on the SiN wall, Na^+ cation and Cl^- anion is respectively accumulated and depleted at the interface. As a result, electric double layer is formed, and electroosmotic flow is yielded by being coupled with the applied electric field (Fig. S20d). The simulated current trace profiles during the translocation of *S. salivarius* were given, including the above-mentioned phenomena such as electricity, ion transport, and electro-osmosis.

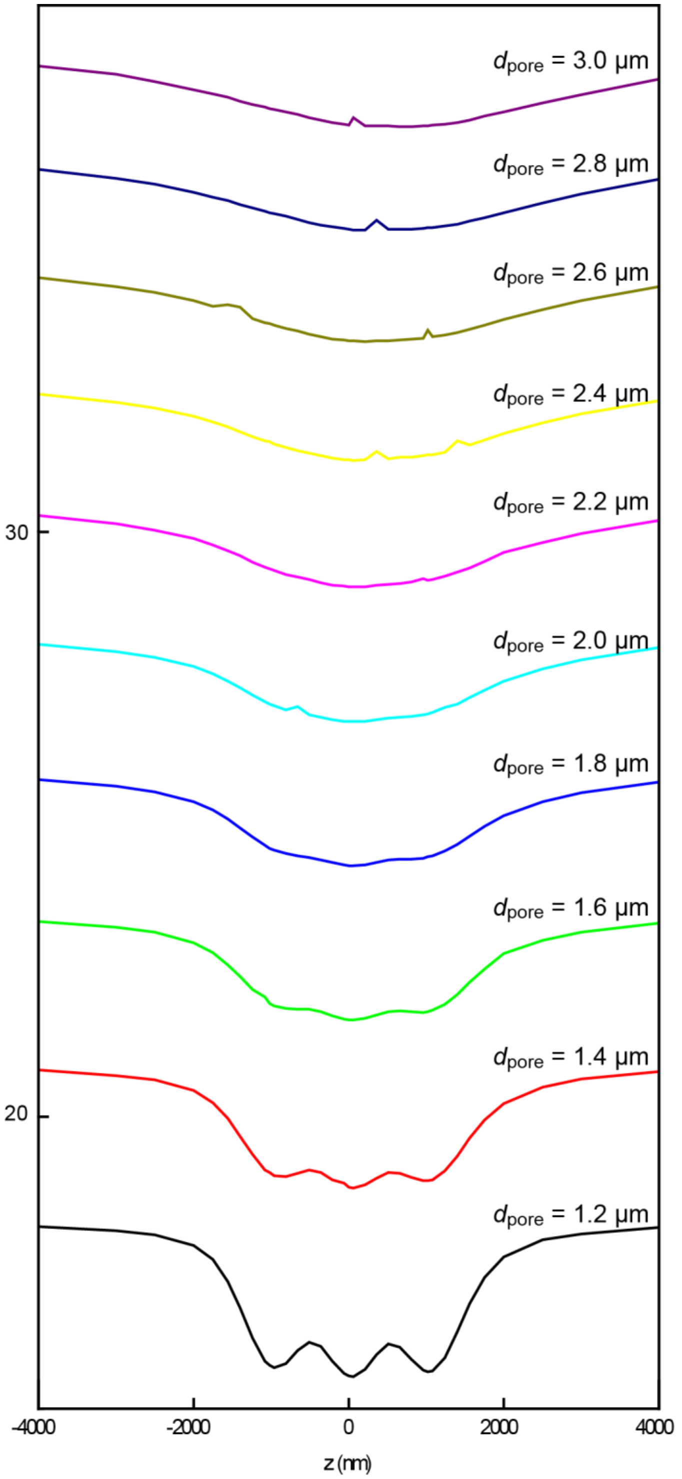

Figure S21. Simulated ionic current spikes for S. salivarius consisting of three cocci. Models of micropores of different diameter d_{pore} ranging from 3.0 μm to 1.2 μm and the same length of L_{pore} = 40 nm were used to calculate the ionic current blockage during axial translocation of individual *S. salivarius* in 0.1 x PBS buffer under the applied

voltage of 0.05 V. Each cocci was sized 0.8 μm and 0.3 μm diameter at the thickest and the thinnest parts, respectively. While the bacterial shape induced corrugation is evident in pores smaller than 1.6 μm, the spikes become increasingly featureless as d_{pore} becomes lager than 1.8 μm due to increasing effects of the access resistance that significantly blurs the ion exclusion effects inside the pore.

3.3 Structural model for *E. coli* and *B. subtilis* for FEM.

Figures S22a and b are the SEM images for *E. coli* and *B. subtilis*. As contrasted with *E. coli* of which appearance is a spheroid-like shape, *B. subtilis* shows a cylinder-like one. To elucidate the shape effect for the measured ionic current trace originating from this shape difference, we constructed the model as follows.

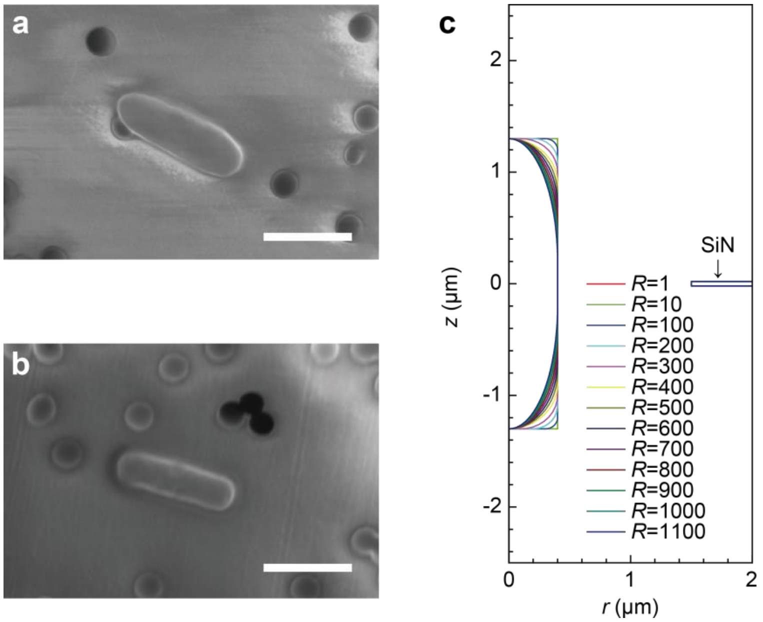

Figure S22. SEM images for **a,** *E. coli* and **b**, *B. subtilis*. Depicted scale bars correspond to 2 μm. **c,** Schematic illustrations for a part of the constructed model for elucidating the difference of shape effect between *E. coli* and *B. subtilis*. Although the size of the modeled *cis*- and *trans*- chambers are 12 (= 4×3) μm radius and 12.12 (= $7\times3^{1/2}$) μm height, the aria around the pore is illustrated. The center position of the object was swept from z = 10 μm to z = − 10 μm, and the case of z = 0 μm is shown.

We used a two-dimensional space with a rotational axis in the same manner as the above-described, and kept the height and radius of the target objects as 2.6 μm and 0.4 μm (Fig. S22c). By filleting the two corner of a rectangle (2.6 μm height and 0.4 μm width) at 1–400 nm radius, we constructed cylinder-like objects which are referred to as R = 1 to R = 400 as shown in Fig. S18c. Regarding the case of $R \geq 400$, we placed the quadrant of the ellipse, of which major- and minor- axis is respectively R nm and 400 nm, at both side of a small rectangle. The height of this small rectangle was adjusted to 2.6 μm − 2R

μm and the width of that was 0.4 μm. There structures are referred to as *R* = 400 to *R* = 1100 in Fig. S18c. Both procedures give the same structure at *R* = 400.

As the pore radius used in the experiment is 1.5 μm, which is larger than that of the pore for *S. salivarius*, we prepared *cis*- and *trans*- chambers as 12 (= 4×3) μm radius and 12.12 (= $7\times3^{1/2}$) μm height. In the simulation, we set a of −15mC/m^2 at the objects surface, and performed the calculation in the same methodology. By sweeping the center position of the objects from *z* = 10 μm to *z* = −10 μm, we obtain the ionic current traces during passing through the objects via the pore. The physical description for the FEM simulations is used in the same manner.

a

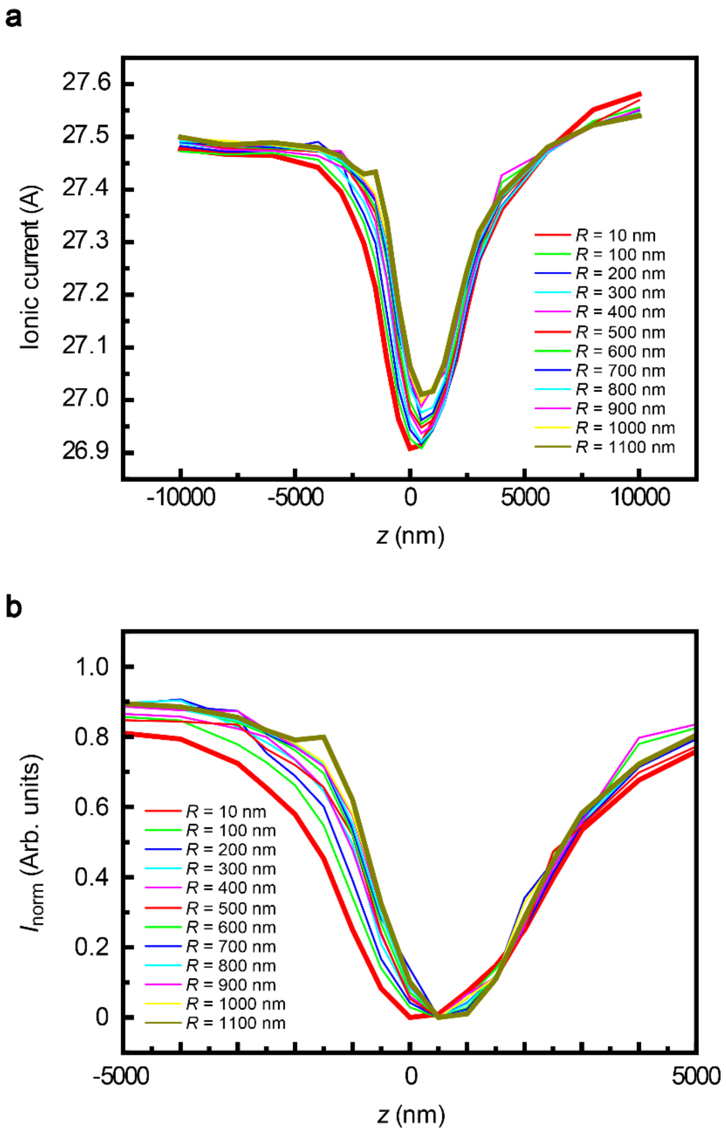

Figure S23. Particle shape-dependence of the ionic spike waveforms. a, Ionic spikes obtained through COMSOL simulations of single-rod-shaped object through a micropore of diameter 3 μm and depth 40 nm. The object has different roundness defined by *R*. Axis-translocation is assumed. Curves for *R* = 10 nm (Black: sharp-edged cylinder) and *R* = 1100 nm (Dark yellow: rounded cylinder) are emphasized for the sake of clarity. **b,** The ionic current curves normalized by the pulse height. Color coding is the same as that in (a).

3.4 Electrophoresis of bacteria

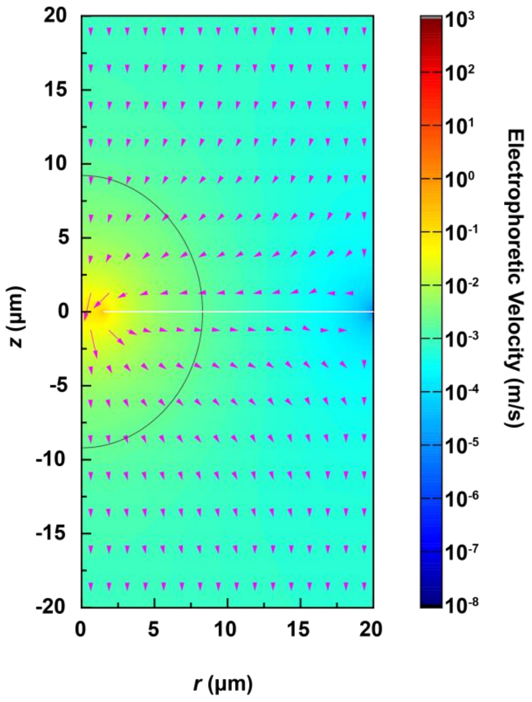

Figure S24. Adsorption radius of bacteria. The electrophoretic velocity of *E. coli* around a micropore of diameter 3 μm and thickness 40 nm under the voltage of 0.05 V. Solid curve illustrates the adsorption radius. The bacteria tend to be drawn into the pore electrophoretically once crossing the boundary.

It is important to clarify whether *E. coli* and *B. subtilis* are allowed to pass through the micropore under the conditions tested.[S4] To elucidate the electrophoretic motion of bacteria, we performed the FEM simulations on the nanopore model of which pore diameter and depth were 3 μm and 40 nm, and also the cell radius and height for chambers of 20 μm were set to prepare enough space in simulating the movement induced by electrophoretic drag force. We simplified the bacterial structure to a sphere of which radius was 0.62 μm, because the volume of *E. coli* and *B. subtilis* was ~1 μm^3. At a steady state, the drag force to an r_s radius sphere on an electrophoretic velocity of v_{ep} is given by $6\pi \mu r_s v_{ep}$, where μ is a dynamic viscosity of fluid. When the spherical particle possesses a charge of q and is placed on an electric field of E, an electrostatic force of qE, which

balances with the drag force, is yielded. By substituting q with $4\pi r_s^2\sigma_s$, here σ_s is the surface charge of the particle, the electrophoretic velocity is expressed as

$$v_{\mathrm{ep}} = \frac{2r_{\mathrm{s}}\sigma_{\mathrm{s}}E}{3\mu}.$$

Figure S25 shows the results of velocity (color map) and velocity field (magenta arrows) of the simulated electrophoresis by using $\sigma_s = -15$ mC/m^2. Along with the electric field, the electrophoresis is yielded between the ground (top side) and the bias voltage terminal (bottom side) via the pore, especially the electrophoretic motion is enhanced around pore.

Another factor regarding the motion of particle dispersed in a fluid is Brownian motion. For the detection of particles, the target particles must be translocated through a pore in finite measurement time by overcoming the Brownian motion. The mean verbosity of Brownian motion v_{BM} is given by

$$\langle v_{\mathrm{BM}} \rangle^2 = \frac{k_{\mathrm{B}}T}{m}.$$

Here, m is the mass of a particle. Assuming the m is the mass of ~1 μm^3 water, v_{BM} = ~2 mm/s is obtained. The green circle in Fig. S25 illustrates the isosurface of $v_{ep} = v_{BM}$, thus the particles are translocated immediately in entering the inside area of the black sphere that extends over 5 μm from the orifice.

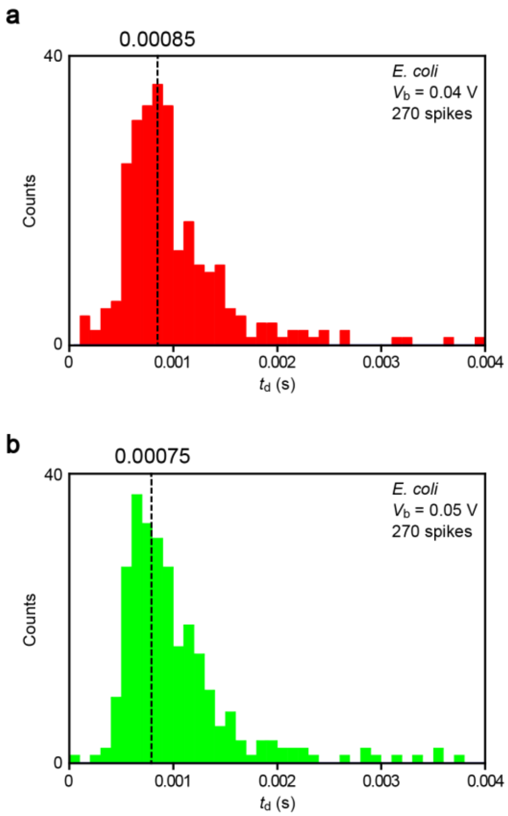

Figure S25. Voltage dependence of translocation time. a-b, Translocation time histograms of *E. coli* measured using a micropore of diameter 3 μm and thickness 40 nm under the applied voltage of 0.04 V (a) and 0.05 V (b).

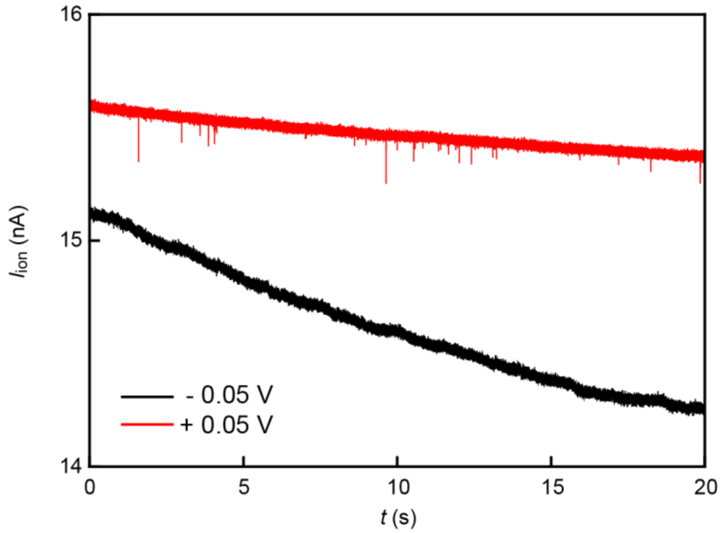

Figure S26. Bias polarity dependent bacterial translocation. The ionic current I_{ion} through a micropore of diameter 3 μm and thickness 40 nm plotted as a function of time. Resistive pulses were observed under positive voltage indicating electrophoretic translocation of the negatively charged *E. coli* (red). In contrast, no signals were detected under negative voltage (black).

4. Supplementary references

[S1] van der Hout, M., Kruddle, V., Janssen, X. J., A., Dekker, N. Distinguishable populations report on the interactions of single-DNA molecules with solid-state nanopores. *Biophys. J.* **99**, 3840-3848 (2010).

[S2] Carlsen, A. T., Zahid, O. K., Ruzicka, J., Taylor, E. W., Hall, A. R. Interpreting the conductance blockades of DNA translocations through solid-state nanopores. *ACS Nano* 8, 4754-4760 (2014).

[S3] Tsutsui, M., He, Y., Yokota, K., Arima, A., Hongo, S., Taniguchi, M., Washio, T. & Kawai, T. Particle trajectory-dependence ionic current blockade in low-aspect-ratio pores. *ACS Nano* 10, 803-809 (2015).

[S4] McMullen, A., de Haan, H. W., Tang, J. X. & Stein, D. Stiff filamentous virus translocations through solid-state nanopores. *Nat. Commun.* 5, 4171 (2014).
